# Supplementary material for: Integrating Enhanced Profiling and Chemometrics to Unveil the Potential Markers for Differentiating among the Leaves of Panax ginseng, P. quinquefolius, and P. notoginseng by Ultra-High Performance Liquid Chromatography/Ion Mobility-Quadrupole Time-of-Flight Mass Spectrometry
Source: Molecules. 2022 Aug 29;27(17):5549. doi: 10.3390/molecules27175549 (PMC9458027; doi:10.3390/molecules27175549)
Supplement: Supplementary file 1 [file molecules-27-05549-s001.zip › molecules-1879769-supplementary.pdf]

<*Molecules*>

Supporting information

Integrating Enhanced Profiling and Chemometrics to Unveil the Potential Markers for Differentiating Among the Leaves of *Panax ginseng*, *P. quinquefolius* and *P. notoginseng* by Ultra-high Performance Liquid Chromatography/Ion Mobility-Quadrupole Time-of-flight Mass Spectrometry

Feifei Yang [1,2,†], Boxue Chen [1,2,†], Meiting Jiang [1,2,†], Huimin Wang[1,2], Ying Hu[1], Hongda Wang[1,2], Xiaoyan Xu[1,2], Xiumei Gao[1,2,3], Wenzhi Yang[1,2,3,*]

[1] State Key Laboratory of Component-based Chinese Medicine, Tianjin University of Traditional Chinese Medicine, 10 Poyanghu Road, Jinghai, Tianjin 301617, China

[2] Haihe Laboratory of Modern Chinese Medicine, Tianjin University of Traditional Chinese Medicine, 10 Poyanghu Road, Jinghai, Tianjin 301617, China

[3] Key Laboratory of Pharmacology of Traditional Chinese Medical Formulae, Ministry of Education, Tianjin University of Traditional Chinese Medicine, 10 Poyanghu Road, Jinghai, Tianjin 301617, China

[*] Correspondence: wzyang0504@tjutcm.edu.cn, Tel.: +86-022-5979-1833 (W.Y.)

[†] These authors contributed equally to this work.

Contents

Figure S1 Chemical structures of 60 reference compounds of ginsenosides (**PPT**: protopanaxatriol; **PPD**: protopanaxadiol; **OA**: oleanolic acid; **OT**: ocotillol).

Figure S2 Method development of the UHPLC/IM-QTOF-MS approach by comparing the influence of column temperature (25–40°C) on the separation of ginsenosides from the QC1 sample, by using the BEH Shield RP18 column.

Figure S3 Method development of the UHPLC/IM-QTOF-MS approach by optimizing ramp collision energy (RCE) for HDMS^E of the hybrid scan approach on the Vion IM-QTOF mass spectrometer, to characterize ginsenosides in the negative mode.

Figure S4 Method development of the UHPLC/IM-QTOF-MS approach by optimizing mass-dependent ramp collision energy (MDRCE) for HDDDA of the hybrid scan approach on the Vion IM-QTOF mass spectrometer, to characterize ginsenosides in the negative mode.

Figure S5 PCA score plot for the multivariate statistical analysis of 33 batches of leaf samples representative of PGL, PQL, and PNL.

Table S1 Information of the 33 batches of the leaf samples for *Panax ginseng*, *Panax quinquefolius*, and *Panax notoginseng* analyzed in this work.

Table S2 Information for 60 ginsenoside reference compounds used in this work.

Table S3 Detailed information for the precursor ions list (PIL) used in the data acquisition approach of the hybrid scan approach.

Table S4 Detailed information of the 342 ginsenoside compounds characterized from the leaves of *Panax ginseng*, *Panax quinquefolius*, and *Panax notoginseng*.

Table S5 Detailed information of 42 potential ginsenoside markers diagnostic for differentiating among PGL, PQL, and PNL.

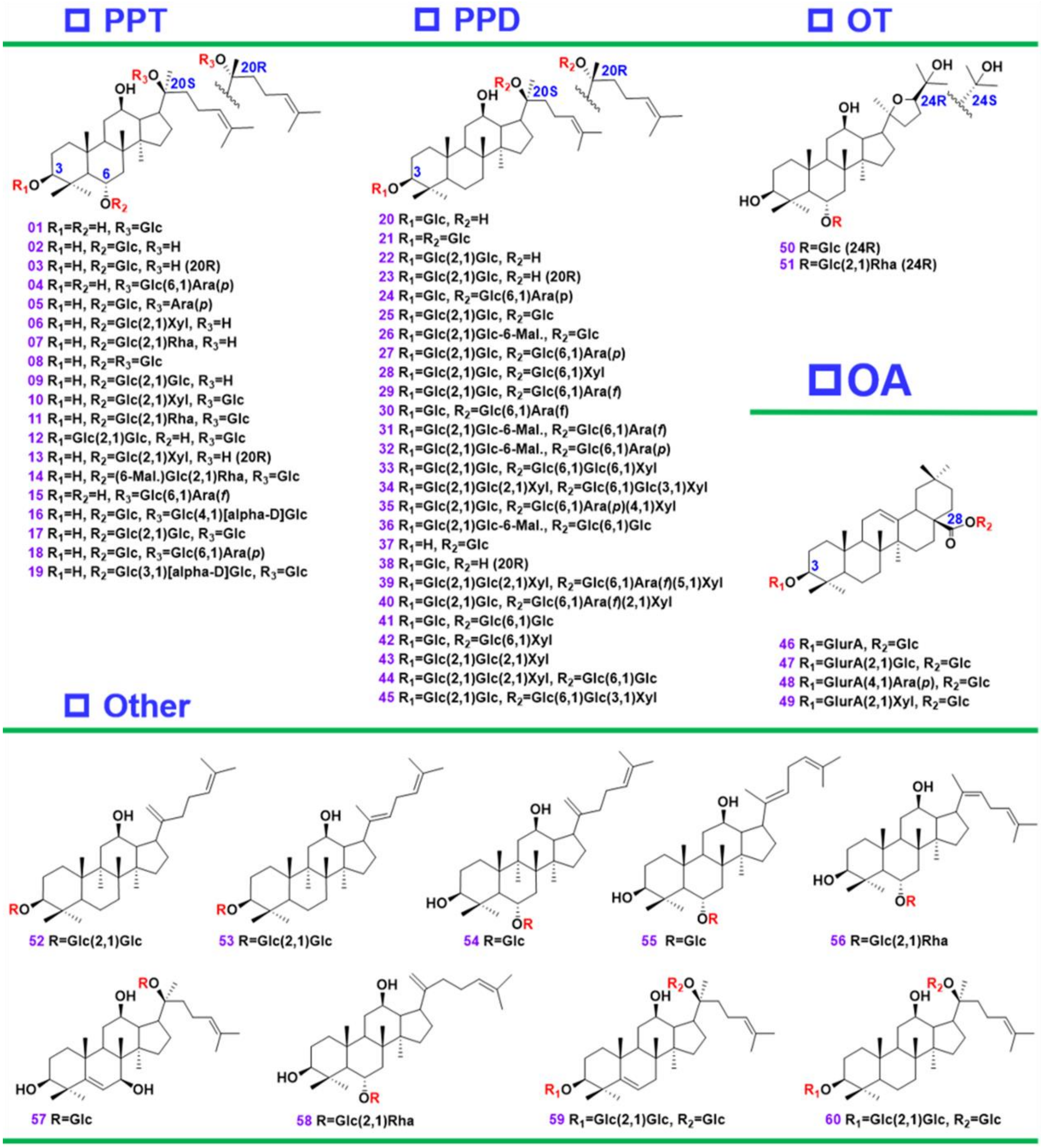

Figure S1 Chemical structures of 60 reference compounds of ginsenosides (**PPT**: protopanaxatriol; **PPD**: protopanaxadiol; **OA**: oleanolic acid; **OT**: ocotillol).

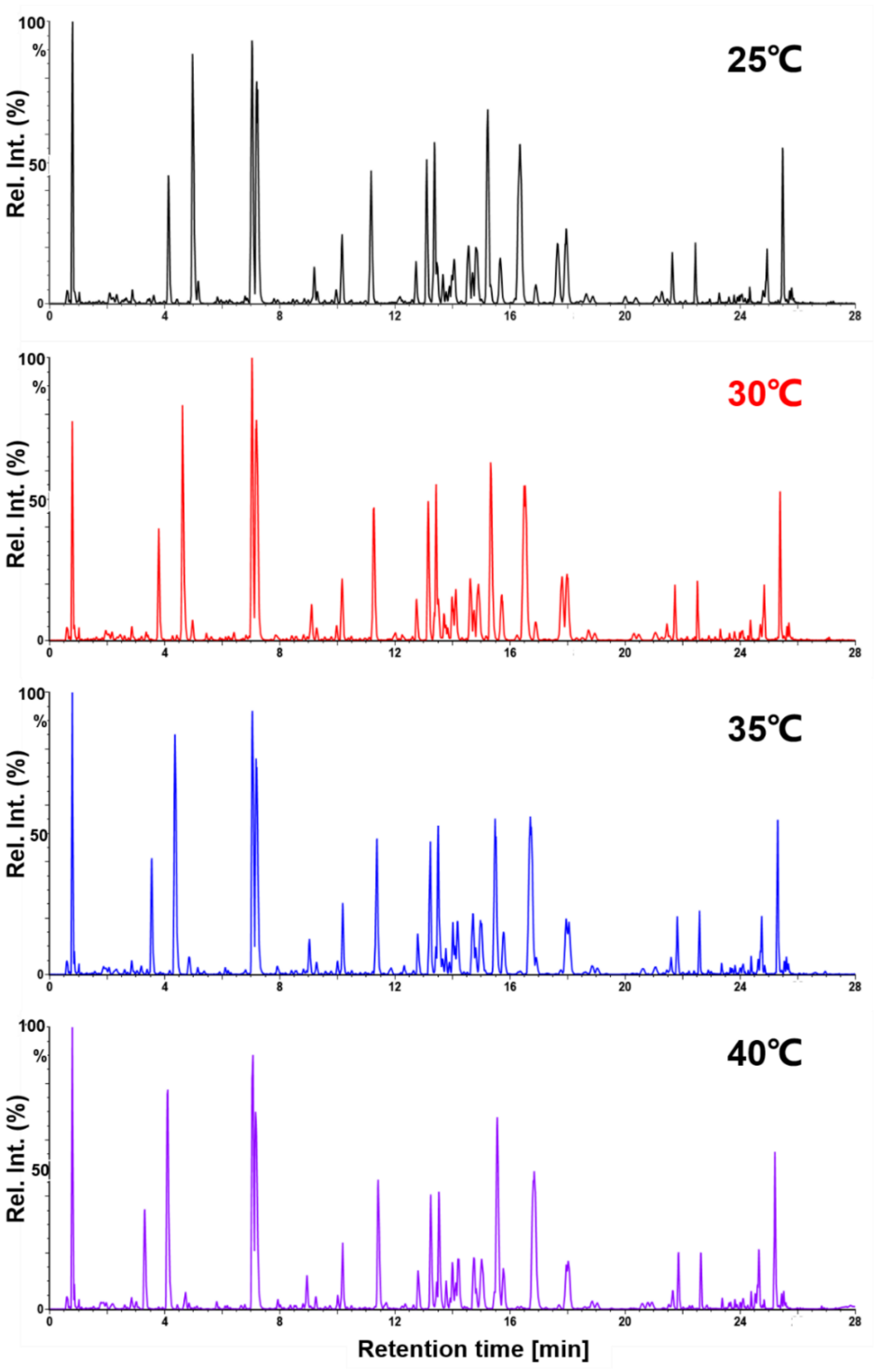

Figure S2 Method development of the UHPLC/IM-QTOF-MS approach by comparing the influence of column temperature (25–40°C) on the separation of ginsenosides from the QC1 sample, by using the BEH Shield RP18 column.

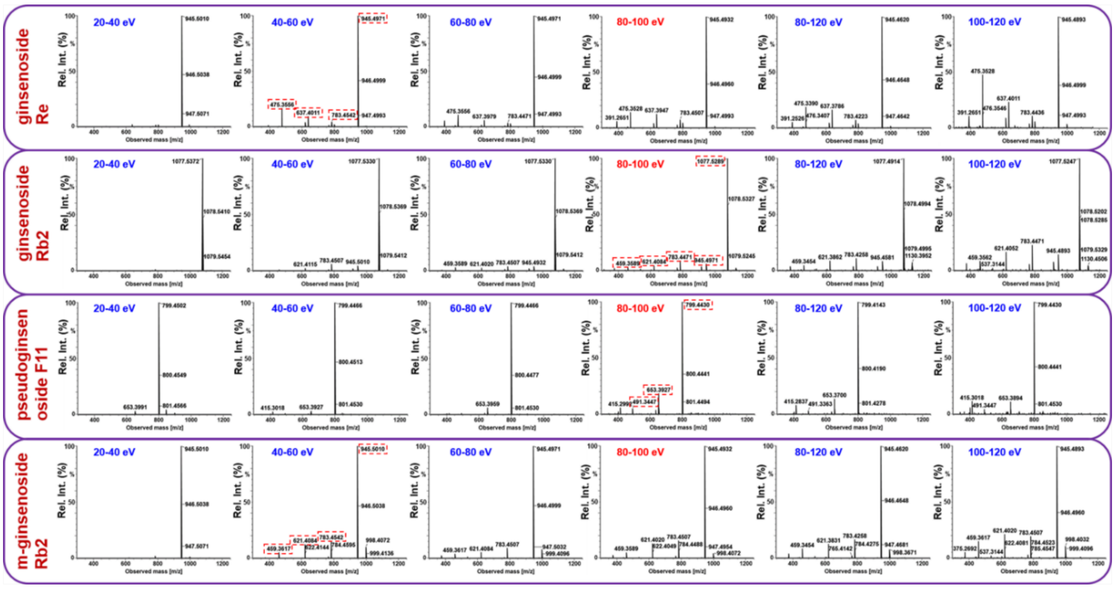

Figure S3 Method development of the UHPLC/IM-QTOF-MS approach by optimizing ramp collision energy (RCE) for HDMS^E of the hybrid scan approach on the Vion IM-QTOF mass spectrometer, to characterize ginsenosides in the negative mode.

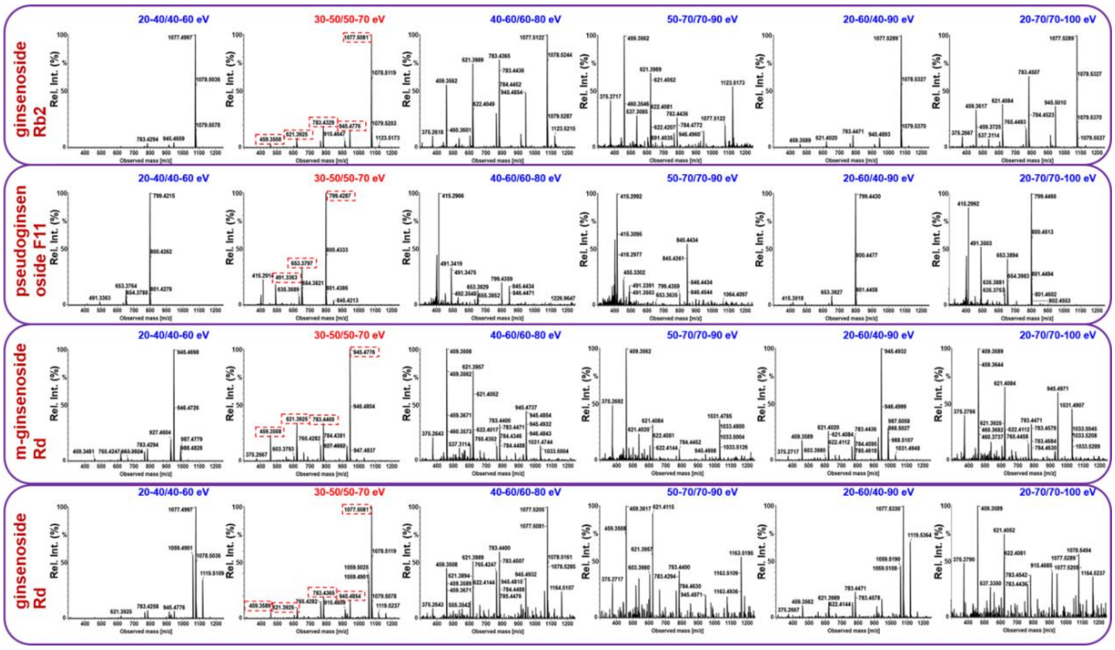

Figure S4 Method development of the UHPLC/IM-QTOF-MS approach by optimizing mass-dependent ramp collision energy (MDRCE) for HDDDA of the hybrid scan approach on the Vion IM-QTOF mass spectrometer, to characterize ginsenosides in the negative mode.

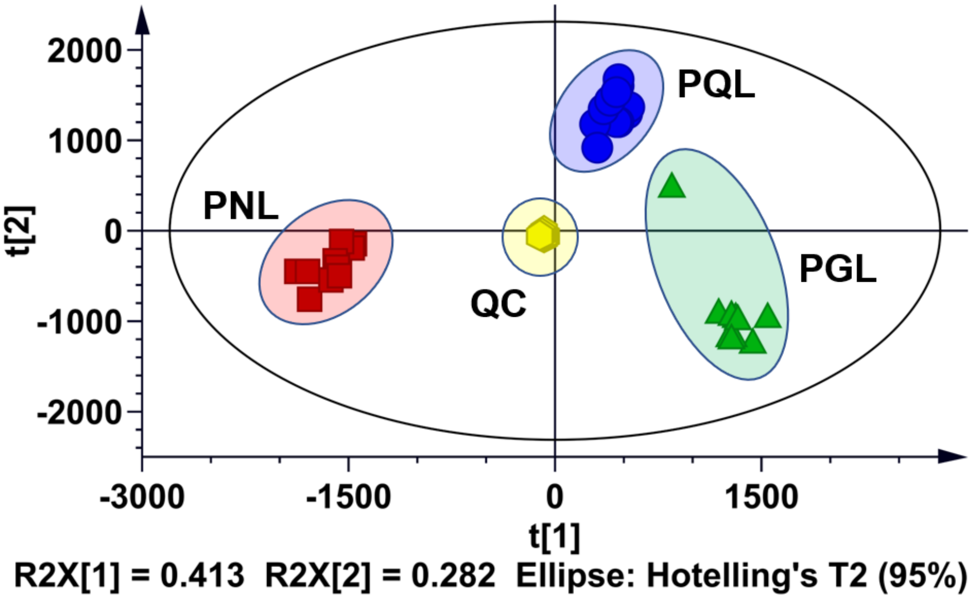

Figure S5 PCA score plot for the multivariate statistical analysis of 33 batches of leaf samples representative of PGL, PQL, and PNL.

Table S1 Information of the 33 batches of the leaf samples for *Panax ginseng*, *Panax quinquefolius*, and *Panax notoginseng* analyzed in this work.

No.	Label	Producing Region	Collection Time
**1**	PQ-1	Baishan City, Jilin Province	2018.10
**2**	PQ-2	Baishan City, Jilin Province	2018.10
**3**	PQ-3	Baishan City, Jilin Province	2018.10
**4**	PQ-4	Baishan City, Jilin Province	2018.10
**5**	PQ-5	Baishan City, Jilin Province	2018.10
**6**	PQ-6	Baishan City, Jilin Province	2018.10
**7**	PQ-7	Baishan City, Jilin Province	2018.10
**8**	PQ-8	Baishan City, Jilin Province	2018.10
**9**	PQ-9	Baishan City, Jilin Province	2018.10
**10**	PQ-10	Baishan City, Jilin Province	2018.10
**11**	PQ-11	Baishan City, Jilin Province	2018.10
**12**	PN-1	Wenshan City, YunNan Province	2018.09
**13**	PN-2	Wenshan City, YunNan Province	2018.09
**14**	PN-3	Wenshan City, YunNan Province	2018.09
**15**	PN-4	Wenshan City, YunNan Province	2018.09
**16**	PN-5	Wenshan City, YunNan Province	2018.09
**17**	PN-6	Wenshan City, YunNan Province	2018.09
**18**	PN-7	Wenshan City, YunNan Province	2018.09
**19**	PN-8	Wenshan City, YunNan Province	2018.09
**20**	PN-9	Wenshan City, YunNan Province	2018.09
**21**	PN-10	Wenshan City, YunNan Province	2018.09
**22**	PN-11	Wenshan City, YunNan Province	2018.09
**23**	PG-1	Baishan City, Jilin Province	2018.09
**24**	PG-2	Baishan City, Jilin Province	2018.09
**25**	PG-3	Baishan City, Jilin Province	2018.09
**26**	PG-4	Baishan City, Jilin Province	2018.09
**27**	PG-5	Baishan City, Jilin Province	2018.09
**28**	PG-6	Baishan City, Jilin Province	2018.09
**29**	PG-7	Baishan City, Jilin Province	2018.09
**30**	PG-8	Baishan City, Jilin Province	2018.09
**31**	PG-9	Baishan City, Jilin Province	2018.09
**32**	PG-10	Baishan City, Jilin Province	2018.09
**33**	PG-11	Baishan City, Jilin Province	2018.09

Table S2 Information for 60 ginsenoside reference compounds used in this work.

No.	Trivial name	M.F.	Exact Mass	Subclass
**1**	ginsenoside F1	$C_{36}H_{62}O_{9}$	638.4394	
**2**	ginsenoside Rh1	$C_{36}H_{62}O_{9}$	638.4394	
**3**	20(*R*)-ginsenoside Rh1	$C_{36}H_{62}O_{9}$	638.4394	
**4**	ginsenoside F3	$C_{41}H_{70}O_{13}$	770.4816	
**5**	20(*S*)-sanchirhinoside A_3	$C_{41}H_{70}O_{13}$	770.4816	
**6**	notoginsenoside R2	$C_{41}H_{70}O_{13}$	770.4816	
**7**	ginsenoside Rg2	$C_{42}H_{72}O_{13}$	784.4973	
**8**	ginsenoside Rg1	$C_{42}H_{72}O_{14}$	800.4922	
**9**	ginsenoside Rf	$C_{42}H_{72}O_{14}$	800.4922	
**10**	notoginsenoside R1	$C_{47}H_{80}O_{18}$	932.5345	**PPT**
**11**	ginsenoside Re	$C_{48}H_{82}O_{18}$	946.5501	
**12**	vinaginsenoside R4	$C_{48}H_{82}O_{19}$	962.5450	
**13**	20(*R*)-notoginsenoside R2	$C_{41}H_{70}O_{13}$	770.4816	
**14**	malonyl-floralginsenoside Re1	$C_{51}H_{84}O_{21}$	1032.5505	
**15**	ginsenoside F5	$C_{41}H_{70}O_{13}$	770.4816	
**16**	ginsenoside Re3	$C_{48}H_{82}O_{19}$	962.5450	
**17**	20-*O*-glucosyl-ginsenoside Rf	$C_{48}H_{82}O_{19}$	962.5450	
**18**	notoginsenoside Fp1	$C_{47}H_{80}O_{18}$	932.5345	
**19**	ginsenoside Re2	$C_{48}H_{82}O_{19}$	962.5450	
**20**	ginsenoside Rh2	$C_{36}H_{62}O_{8}$	622.4445	
**21**	ginsenoside F2	$C_{42}H_{72}O_{13}$	784.4973	
**22**	20(*S*)-ginsenoside Rg3	$C_{42}H_{72}O_{13}$	784.4973	
**23**	20(*R*)-ginsenoside Rg3	$C_{42}H_{72}O_{13}$	784.4973	
**24**	ginsenoside Rd2	$C_{47}H_{80}O_{17}$	916.5396	
**25**	ginsenoside Rd	$C_{48}H_{82}O_{18}$	946.5501	
**26**	malonyl-ginsenoside Rd	$C_{51}H_{84}O_{21}$	1032.5505	
**27**	ginsenoside Rb2	$C_{53}H_{90}O_{22}$	1078.5924	**PPD**
**28**	ginsenoside Rb3	$C_{53}H_{90}O_{22}$	1078.5924	
**29**	ginsenoside Rc	$C_{53}H_{90}O_{22}$	1078.5924	
**30**	notoginsenoside Fe	$C_{47}H_{80}O_{17}$	916.5396	
**31**	malonyl-ginsenoside Rc	$C_{56}H_{92}O_{25}$	1164.5928	
**32**	malonyl-ginsenoside Rb2	$C_{56}H_{92}O_{25}$	1164.5928	
**33**	notoginsenoside R4	$C_{59}H_{100}O_{27}$	1240.6452	

**34**	notoginsenoside T	$C_{64}H_{108}O_{31}$	1372.6875	
**35**	ginsenoside Ra1	$C_{58}H_{98}O_{26}$	1210.6346	
**36**	malonyl-ginsenoside Rb1	$C_{57}H_{94}O_{26}$	1194.6033	
**37**	compound K	$C_{36}H_{62}O_{8}$	622.4445	
**38**	20(*R*)-ginsenoside Rh2	$C_{36}H_{62}O_{8}$	622.4445	
**39**	notoginsenoside S	$C_{63}H_{106}O_{30}$	1342.6769	
**40**	ginsenoside Ra2	$C_{58}H_{98}O_{26}$	1210.6346	
**41**	gypenoside XVII	$C_{48}H_{82}O_{18}$	946.5501	
**42**	notoginsenoside Fd	$C_{47}H_{80}O_{17}$	916.5396	
**43**	notoginsenoside Ft1	$C_{47}H_{80}O_{17}$	916.5396	
**44**	notoginsenoside Fa	$C_{59}H_{100}O_{27}$	1240.6452	
**45**	ginsenoside Ra3	$C_{59}H_{100}O_{27}$	1240.6452	
**46**	chikusetsusaponin IVa	$C_{42}H_{66}O_{14}$	794.4453	**OA**
**47**	ginsenoside Ro	$C_{48}H_{76}O_{19}$	956.4981	
**48**	chikusetsusaponin IV	$C_{47}H_{74}O_{18}$	926.4875	
**49**	pseudoginsenoside Rt1	$C_{47}H_{74}O_{18}$	926.4875	
**50**	24(*R*)-pseudoginsenoside Rt5	$C_{36}H_{62}O_{10}$	654.4343	**OT**
**51**	24(*R*)-pseudoginsenoside F11	$C_{42}H_{72}O_{14}$	800.4922	
**52**	ginsenoside Rk1	$C_{42}H_{70}O_{12}$	766.4868	**Other**
**53**	ginsenoside Rg5	$C_{42}H_{70}O_{12}$	766.4868	
**54**	ginsenoside Rk3	$C_{36}H_{60}O_{8}$	620.4288	
**55**	ginsenoside Rh4	$C_{36}H_{60}O_{8}$	620.4288	
**56**	(20*E*)-ginsenoside F4	$C_{42}H_{70}O_{12}$	766.4867	
**57**	ginsenoside Rh7	$C_{36}H_{60}O_{9}$	636.4237	
**58**	ginsenoside Rg6	$C_{42}H_{70}O_{12}$	766.4867	
**59**	5,6-didehydroginsenoside Rd	$C_{48}H_{80}O_{18}$	944.5345	
**60**	vinaginsenoside R8	$C_{48}H_{82}O_{19}$	962.5450	

Table S3 Detailed information for the precursor ions list (PIL) used in the data acquisition approach of the hybrid scan approach.

No.	Mass [*m/z*]	Formula [M]	Formula type	Precursor Ion Species	Polarity
1	437.2909	$C_{24}H_{40}O_4$	Chemical formula	+HCOO	Negative
2	503.3378	$C_{29}H_{46}O_4$	Chemical formula	+HCOO	Negative
3	503.3742	$C_{30}H_{50}O_3$	Chemical formula	+HCOO	Negative
4	505.3899	$C_{30}H_{52}O_3$	Chemical formula	+HCOO	Negative
5	517.3535	$C_{30}H_{48}O_4$	Chemical formula	+HCOO	Negative
6	519.3691	$C_{30}H_{50}O_4$	Chemical formula	+HCOO	Negative
7	521.3848	$C_{30}H_{52}O_4$	Chemical formula	+HCOO	Negative
8	523.4004	$C_{30}H_{54}O_4$	Chemical formula	+HCOO	Negative
9	533.3484	$C_{30}H_{48}O_5$	Chemical formula	+HCOO	Negative
10	535.3640	$C_{30}H_{50}O_5$	Chemical formula	+HCOO	Negative
11	537.3797	$C_{30}H_{52}O_5$	Chemical formula	+HCOO	Negative
12	537.4161	$C_{31}H_{56}O_4$	Chemical formula	+HCOO	Negative
13	549.4161	$C_{32}H_{56}O_4$	Chemical formula	+HCOO	Negative
14	551.4317	$C_{32}H_{58}O_4$	Chemical formula	+HCOO	Negative
15	553.3746	$C_{30}H_{52}O_6$	Chemical formula	+HCOO	Negative
16	553.4110	$C_{31}H_{56}O_5$	Chemical formula	+HCOO	Negative
17	563.3953	$C_{32}H_{54}O_5$	Chemical formula	+HCOO	Negative
18	583.3488	$C_{30}H_{50}O_8$	Chemical formula	+HCOO	Negative
19	599.3437	$C_{30}H_{50}O_9$	Chemical formula	+HCOO	Negative
20	611.3801	$C_{32}H_{54}O_8$	Chemical formula	+HCOO	Negative
21	617.4059	$C_{36}H_{58}O_8$	Chemical formula	–H	Negative
22	619.4427	$C_{32}H_{62}O_8$	Chemical formula	+HCOO	Negative

No.	Mass [*m/z*]	Formula [M]	Formula type	Precursor Ion Species	Polarity
23	625.3593	$C_{32}H_{52}O_9$	Chemical formula	+HCOO	Negative
24	633.4008	$C_{36}H_{58}O_9$	Chemical formula	–H	Negative
25	635.4165	$C_{35}H_{58}O_7$	Chemical formula	+HCOO	Negative
26	635.5409	$C_{42}H_{70}O$	Chemical formula	+HCOO	Negative
27	649.4321	$C_{36}H_{60}O_7$	Chemical formula	+HCOO	Negative
28	663.4266	$C_{40}H_{58}O_5$	Chemical formula	+HCOO	Negative
29	665.4270	$C_{36}H_{60}O_8$	Chemical formula	+HCOO	Negative
30	666.3985	$C_{35}H_{57}O_9$	Chemical formula	+HCOO	Negative
31	667.4427	$C_{36}H_{62}O_8$	Chemical formula	+HCOO	Negative
32	681.4219	$C_{36}H_{60}O_9$	Chemical formula	+HCOO	Negative
33	683.4376	$C_{36}H_{62}O_9$	Chemical formula	+HCOO	Negative
34	687.4478	$C_{40}H_{64}O_9$	Chemical formula	–H	Negative
35	689.4270	$C_{38}H_{60}O_8$	Chemical formula	+HCOO	Negative
36	695.4012	$C_{36}H_{58}O_{10}$	Chemical formula	+HCOO	Negative
37	697.4169	$C_{36}H_{60}O_{10}$	Chemical formula	+HCOO	Negative
38	697.4532	$C_{37}H_{64}O_9$	Chemical formula	+HCOO	Negative
39	699.4325	$C_{36}H_{62}O_{10}$	Chemical formula	+HCOO	Negative
40	701.4482	$C_{36}H_{64}O_{10}$	Chemical formula	+HCOO	Negative
41	703.4274	$C_{35}H_{62}O_{11}$	Chemical formula	+HCOO	Negative
42	707.4376	$C_{38}H_{62}O_9$	Chemical formula	+HCOO	Negative
43	709.4532	$C_{38}H_{64}O_9$	Chemical formula	+HCOO	Negative
44	711.4325	$C_{37}H_{62}O_{10}$	Chemical formula	+HCOO	Negative
45	713.4285	$C_{37}H_{64}O_{10}$	Chemical formula	+ HCOO	Negative
46	713.4482	$C_{37}H_{64}O_{10}$	Chemical formula	+ HCOO	Negative
47	715.4274	$C_{36}H_{62}O_{11}$	Chemical formula	+HCOO	Negative

No.	Mass [*m/z*]	Formula [M]	Formula type	Precursor Ion Species	Polarity
48	717.4431	$C_{36}H_{64}O_{11}$	Chemical formula	+HCOO	Negative
49	725.4482	$C_{38}H_{64}O_{10}$	Chemical formula	+HCOO	Negative
50	729.4431	$C_{37}H_{64}O_{11}$	Chemical formula	+HCOO	Negative
51	747.4325	$C_{41}H_{64}O_{12}$	Chemical formula	–H	Negative
52	748.5495	$C_{43}H_{75}O_{7}$	Chemical formula	+HCOO	Negative
53	751.4638	$C_{40}H_{66}O_{10}$	Chemical formula	+HCOO	Negative
54	763.4274	$C_{41}H_{64}O_{13}$	Chemical formula	–H	Negative
55	763.4638	$C_{42}H_{68}O_{12}$	Chemical formula	–H	Negative
56	765.5675	$C_{48}H_{78}O_{7}$	Chemical formula	–H	Negative
57	767.4587	$C_{40}H_{66}O_{11}$	Chemical formula	+HCOO	Negative
58	769.5988	$C_{47}H_{80}O_{5}$	Chemical formula	+HCOO	Negative
59	771.4172	$C_{38}H_{62}O_{13}$	Chemical formula	+HCOO	Negative
60	777.4431	$C_{42}H_{66}O_{13}$	Chemical formula	–H	Negative
61	781.4744	$C_{41}H_{68}O_{11}$	Chemical formula	+HCOO	Negative
62	783.4536	$C_{40}H_{66}O_{12}$	Chemical formula	+HCOO	Negative
63	785.4693	$C_{40}H_{68}O_{12}$	Chemical formula	+HCOO	Negative
64	787.4849	$C_{40}H_{70}O_{12}$	Chemical formula	+HCOO	Negative
65	793.4380	$C_{42}H_{66}O_{14}$	Chemical formula	–H	Negative
66	797.4693	$C_{41}H_{68}O_{12}$	Chemical formula	+HCOO	Negative
67	799.4849	$C_{41}H_{70}O_{12}$	Chemical formula	+HCOO	Negative
68	801.4642	$C_{40}H_{68}O_{13}$	Chemical formula	+HCOO	Negative
69	807.4536	$C_{43}H_{68}O_{14}$	Chemical formula	–H	Negative
70	811.4849	$C_{42}H_{70}O_{12}$	Chemical formula	+HCOO	Negative
71	815.4798	$C_{41}H_{70}O_{13}$	Chemical formula	+HCOO	Negative
72	827.4798	$C_{42}H_{70}O_{13}$	Chemical formula	+HCOO	Negative

No.	Mass [*m/z*]	Formula [M]	Formula type	Precursor Ion Species	Polarity
73	829.4955	$C_{42}H_{72}O_{13}$	Chemical formula	+HCOO	Negative
74	830.5033	$C_{42}H_{73}O_{13}$	Chemical formula	+HCOO	Negative
75	831.4748	$C_{41}H_{70}O_{14}$	Chemical formula	+HCOO	Negative
76	843.4748	$C_{42}H_{70}O_{14}$	Chemical formula	+HCOO	Negative
77	843.5111	$C_{43}H_{74}O_{13}$	Chemical formula	+HCOO	Negative
78	844.4826	$C_{42}H_{71}O_{14}$	Chemical formula	+HCOO	Negative
79	845.4904	$C_{42}H_{72}O_{14}$	Chemical formula	+HCOO	Negative
80	847.4697	$C_{41}H_{70}O_{15}$	Chemical formula	+HCOO	Negative
81	847.5061	$C_{42}H_{74}O_{14}$	Chemical formula	+HCOO	Negative
82	849.5006	$C_{46}H_{74}O_{14}$	Chemical formula	–H	Negative
83	853.4955	$C_{44}H_{72}O_{13}$	Chemical formula	+HCOO	Negative
84	857.4904	$C_{43}H_{72}O_{14}$	Chemical formula	+HCOO	Negative
85	859.4697	$C_{42}H_{70}O_{15}$	Chemical formula	+HCOO	Negative
86	861.4853	$C_{42}H_{72}O_{15}$	Chemical formula	+HCOO	Negative
87	863.5010	$C_{42}H_{74}O_{15}$	Chemical formula	+HCOO	Negative
88	871.5061	$C_{44}H_{74}O_{14}$	Chemical formula	+HCOO	Negative
89	873.4853	$C_{43}H_{72}O_{15}$	Chemical formula	+HCOO	Negative
90	875.5010	$C_{43}H_{74}O_{15}$	Chemical formula	+HCOO	Negative
91	877.4802	$C_{42}H_{72}O_{16}$	Chemical formula	+HCOO	Negative
92	879.4959	$C_{42}H_{74}O_{16}$	Chemical formula	+HCOO	Negative
93	887.5010	$C_{44}H_{74}O_{15}$	Chemical formula	+HCOO	Negative
94	903.4959	$C_{44}H_{74}O_{16}$	Chemical formula	+HCOO	Negative
95	913.5166	$C_{46}H_{76}O_{15}$	Chemical formula	+HCOO	Negative
96	925.4802	$C_{47}H_{74}O_{18}$	Chemical formula	–H	Negative
97	939.4959	$C_{48}H_{76}O_{18}$	Chemical formula	–H	Negative

No.	Mass [*m/z*]	Formula [M]	Formula type	Precursor Ion Species	Polarity
98	941.5115	$C_{48}H_{78}O_{18}$	Chemical formula	–H	Negative
99	947.5221	$C_{46}H_{78}O_{17}$	Chemical formula	+HCOO	Negative
100	953.5115	$C_{49}H_{78}O_{18}$	Chemical formula	–H	Negative
101	955.4908	$C_{48}H_{76}O_{19}$	Chemical formula	–H	Negative
102	958.5507	$C_{48}H_{81}O_{16}$	Chemical formula	+HCOO	Negative
103	959.5221	$C_{47}H_{78}O_{17}$	Chemical formula	+HCOO	Negative
104	961.5378	$C_{47}H_{80}O_{17}$	Chemical formula	+HCOO	Negative
105	965.4963	$C_{45}H_{76}O_{19}$	Chemical formula	+HCOO	Negative
106	969.5065	$C_{49}H_{78}O_{19}$	Chemical formula	–H	Negative
107	975.5534	$C_{48}H_{82}O_{17}$	Chemical formula	+HCOO	Negative
108	977.5327	$C_{47}H_{80}O_{18}$	Chemical formula	+HCOO	Negative
109	981.4912	$C_{45}H_{76}O_{20}$	Chemical formula	+HCOO	Negative
110	981.5428	$C_{51}H_{82}O_{18}$	Chemical formula	–H	Negative
111	983.5221	$C_{50}H_{80}O_{19}$	Chemical formula	–H	Negative
112	989.5327	$C_{48}H_{80}O_{18}$	Chemical formula	+HCOO	Negative
113	991.5483	$C_{48}H_{82}O_{18}$	Chemical formula	+HCOO	Negative
114	993.5276	$C_{47}H_{80}O_{19}$	Chemical formula	+HCOO	Negative
115	1005.5276	$C_{48}H_{80}O_{19}$	Chemical formula	+HCOO	Negative
116	1007.5432	$C_{48}H_{82}O_{19}$	Chemical formula	+HCOO	Negative
117	1011.5534	$C_{52}H_{84}O_{19}$	Chemical formula	–H	Negative
118	1023.5382	$C_{48}H_{82}O_{20}$	Chemical formula	+HCOO	Negative
119	1025.5538	$C_{48}H_{84}O_{20}$	Chemical formula	+HCOO	Negative
120	1031.5432	$C_{51}H_{84}O_{21}$	Chemical formula	–H	Negative
121	1033.5589	$C_{50}H_{84}O_{19}$	Chemical formula	+HCOO	Negative
122	1039.5331	$C_{48}H_{82}O_{21}$	Chemical formula	+HCOO	Negative

No.	Mass [*m/z*]	Formula [M]	Formula type	Precursor Ion Species	Polarity
123	1049.5538	$C_{50}H_{84}O_{20}$	Chemical formula	+HCOO	Negative
124	1053.6156	$C_{59}H_{90}O_{16}$	Chemical formula	–H	Negative
125	1057.5225	$C_{52}H_{82}O_{22}$	Chemical formula	–H	Negative
126	1059.5745	$C_{52}H_{86}O_{19}$	Chemical formula	+HCOO	Negative
127	1065.5487	$C_{50}H_{84}O_{21}$	Chemical formula	+HCOO	Negative
128	1071.5382	$C_{53}H_{84}O_{22}$	Chemical formula	–H	Negative
129	1083.5136	$C_{49}H_{82}O_{23}$	Chemical formula	+ HCOO	Negative
130	1087.5331	$C_{53}H_{84}O_{23}$	Chemical formula	–H	Negative
131	1091.5643	$C_{53}H_{88}O_{23}$	Chemical formula	+ HCOO	Negative
132	1093.5800	$C_{52}H_{88}O_{21}$	Chemical formula	+HCOO	Negative
133	1101.5487	$C_{54}H_{86}O_{23}$	Chemical formula	–H	Negative
134	1117.5436	$C_{54}H_{86}O_{24}$	Chemical formula	–H	Negative
135	1121.5749	$C_{53}H_{88}O_{22}$	Chemical formula	+HCOO	Negative
136	1123.5906	$C_{53}H_{90}O_{22}$	Chemical formula	+HCOO	Negative
137	1137.5698	$C_{53}H_{88}O_{23}$	Chemical formula	+HCOO	Negative
138	1137.6062	$C_{54}H_{92}O_{22}$	Chemical formula	+HCOO	Negative
139	1138.6141	$C_{54}H_{93}O_{22}$	Chemical formula	+HCOO	Negative
140	1139.5855	$C_{53}H_{90}O_{23}$	Chemical formula	+HCOO	Negative
141	1151.5855	$C_{54}H_{90}O_{23}$	Chemical formula	+HCOO	Negative
142	1153.6011	$C_{54}H_{92}O_{23}$	Chemical formula	+HCOO	Negative
143	1155.5804	$C_{53}H_{90}O_{24}$	Chemical formula	+HCOO	Negative
144	1157.5961	$C_{53}H_{92}O_{24}$	Chemical formula	+HCOO	Negative
145	1163.5855	$C_{56}H_{92}O_{25}$	Chemical formula	–H	Negative
146	1165.6011	$C_{55}H_{92}O_{23}$	Chemical formula	+HCOO	Negative
147	1167.5804	$C_{54}H_{90}O_{24}$	Chemical formula	+HCOO	Negative

No.	Mass [*m/z*]	Formula [M]	Formula type	Precursor Ion Species	Polarity
148	1169.5961	$C_{54}H_{92}O_{24}$	Chemical formula	+HCOO	Negative
149	1172.6559	$C_{55}H_{99}O_{23}$	Chemical formula	+HCOO	Negative
150	1183.8969	$C_{70}H_{122}O_{11}$	Chemical formula	+HCOO	Negative
151	1185.5910	$C_{54}H_{92}O_{25}$	Chemical formula	+HCOO	Negative
152	1185.6579	$C_{64}H_{98}O_{20}$	Chemical formula	–H	Negative
153	1187.6066	$C_{54}H_{94}O_{25}$	Chemical formula	+HCOO	Negative
154	1190.6090	$C_{57}H_{93}O_{23}$	Chemical formula	+HCOO	Negative
155	1191.6168	$C_{57}H_{94}O_{23}$	Chemical formula	+HCOO	Negative
156	1193.5961	$C_{57}H_{94}O_{26}$	Chemical formula	–H	Negative
157	1195.6117	$C_{56}H_{94}O_{24}$	Chemical formula	+HCOO	Negative
158	1197.6274	$C_{56}H_{96}O_{24}$	Chemical formula	+HCOO	Negative
159	1215.5562	$C_{54}H_{90}O_{27}$	Chemical formula	+HCOO	Negative
160	1215.6684	$C_{65}H_{100}O_{21}$	Chemical formula	–H	Negative
161	1221.6274	$C_{58}H_{96}O_{24}$	Chemical formula	+HCOO	Negative
162	1239.6379	$C_{58}H_{98}O_{25}$	Chemical formula	+HCOO	Negative
163	1255.6328	$C_{58}H_{98}O_{26}$	Chemical formula	+HCOO	Negative
164	1269.6485	$C_{59}H_{100}O_{26}$	Chemical formula	+HCOO	Negative
165	1271.6278	$C_{58}H_{98}O_{27}$	Chemical formula	+HCOO	Negative
166	1277.6900	$C_{62}H_{104}O_{24}$	Chemical formula	+HCOO	Negative
167	1285.6434	$C_{59}H_{100}O_{27}$	Chemical formula	+HCOO	Negative
168	1296.6356	$C_{60}H_{99}O_{27}$	Chemical formula	+HCOO	Negative
169	1315.6540	$C_{60}H_{102}O_{28}$	Chemical formula	+HCOO	Negative
170	1323.6591	$C_{62}H_{102}O_{27}$	Chemical formula	+HCOO	Negative
171	1325.6383	$C_{62}H_{102}O_{30}$	Chemical formula	–H	Negative
172	1327.6540	$C_{61}H_{102}O_{28}$	Chemical formula	+HCOO	Negative

No.	Mass [*m/z*]	Formula [M]	Formula type	Precursor Ion Species	Polarity
173	1387.6751	$C_{63}H_{106}O_{30}$	Chemical formula	+HCOO	Negative
174	1417.6857	$C_{64}H_{108}O_{31}$	Chemical formula	+HCOO	Negative

Table S4 Detailed information of the 342 ginsenoside compounds characterized from the leaves of *Panax ginseng*, *Panax quinquefolius*, and *Panax notoginseng*.

No.	t_R (min)	Observed *m/z*	Adduct	Formula	Mass error (ppm)	Observed CCS (Å^2)	Match or not with Database	ESI-MS^2	Identification	Botany Source
**1**	1.63	1025.5540	–H, +HCOO	$C_{48}H_{84}O_{20}$	0.19	325.7827	1	979.5484, 799.4835, 653.4278, 491.3758	ginsengenin-S3 or isomer（OT-2Glc-Rha-H_2O）	PQL、PGL
**2**	2.49	861.4859	–H	$C_{43}H_{74}O_{17}$	0.67	297.2756	0	501.2064, 463.2127, 391.2841	$C_{24}H_{40}O_4$-2Glc-Rha	PGL
**3**	2.51	993.5283	–H, +HCOO	$C_{47}H_{80}O_{19}$	0.73	315.8650	1	947.5312, 785.4709, 765.4589, 639.4084, 477.3567, 459.3448	notoginsenoside H or isomer（$C_{30}H_{52}O_3$-2Glc-Rha-H_2O）	PQL、PGL
**4**	2.74	1007.5436	–H, +HCOO	$C_{48}H_{82}O_{19}$	0.46	326.1775	1	961.5380, 781.4744, 635.4165, 491.3735	quinquenoside L2（OT-2Glc-Rha）	PQL、PGL
**5**	2.92	877.4811	–H	$C_{43}H_{74}O_{18}$	1.06	298.1563	0	651.4095, 633.4003, 471.3478, 391.2832	$C_{24}H_{40}O_4$-3Glc	PGL
**6**	3.16	1007.5421	–H, +HCOO	$C_{48}H_{82}O_{19}$	–1.11	323.6277	1	961.5404, 781.4747, 635.4156, 491.3730	quinquenoside L2（OT-2Glc-Rha）	PGL
**7**	3.27	977.5335	–H	$C_{48}H_{82}O_{20}$	0.94	323.4536	1	977.5237, 765.4432, 619.3842, 471.3471, 391.2844	floralginsenoside I or isomer（$C_{24}H_{40}O_4$-GlurA-2Glc-Bute-H_2O）	PQL、PGL
**8**	3.43	991.5118	–H, +HCOO	$C_{47}H_{78}O_{19}$	–0.09	319.9370	0	945.5065, 765.4428, 619.3872, 457.3313	$C_{30}H_{50}O_3$-2Glc-Rha-H_2O	PGL
**9**	3.50	1005.5272	–H, +HCOO	$C_{48}H_{80}O_{19}$	–0.41	320.5042	1	797.4637, 779.4590, 633.4013, 471.3472, 453.3354, 391.2836	vinaginsenoside R20 or isomer（$C_{24}H_{40}O_4$-GlurA-Glc-Rha-2Ace）	PGL
**10**	3.58	1049.5518	–H, +HCOO	$C_{50}H_{84}O_{20}$	–1.98	333.9221	1	961.5429, 781.4751, 635.4164, 391.2800	6-acetyl ginsenoside Rg3 or isomer（$C_{24}H_{40}O_4$-3Glc-3Ace）	PGL
**11**	3.80	1007.5438	–H, +HCOO	$C_{48}H_{82}O_{19}$	0.63	315.3317	1	961.5371, 815.4790, 797.4697, 653.4255, 491.3755	quinquenoside L2（OT-2Glc-Rha）	PQL
**12**	3.80	717.4433	–H, +HCOO	$C_{36}H_{64}O_{11}$	0.34	264.1698	1	671.4389, 509.3846, 391.2847	vinaginsenoside R12（$C_{24}H_{40}O_4$-GlurA-Mal-H_2O）	PQL
**13**	3.96	1005.5279	–H, +HCOO	$C_{48}H_{80}O_{19}$	0.36	321.3695	1	959.5301, 779.4594, 633.3998, 471.3507, 391.2836	vinaginsenoside R20 or isomer（$C_{24}H_{40}O_4$-GlurA-Glc-Rha-2Ace）	PQL、PGL
**14**	4.05	863.5018	–H, +HCOO	$C_{42}H_{74}O_{15}$	0.95	291.6905	1	817.4963, 671.4375, 653.4265, 509.3845, 391.2847	quinquenoside L9（$C_{24}H_{40}O_4$-2Glc-2Ace-H_2O）	PQL
**15**	4.08	1023.5385	–H, +HCOO	$C_{48}H_{82}O_{20}$	0.45	325.5498	1	977.5311, 797.4711, 653.4185, 491.3727	yesanchinoside B or isomer（OT-3Glc）	PGL
**16**	4.18	855.3311	–H	$C_{40}H_{56}O_{20}$	2.23	273.4744	0	809.3235, 805.2941, 761.3037, 491.1670	OT-Rha-Xyl-Mal	PQL
**17**	4.36	1065.5492	–H, +HCOO	$C_{50}H_{84}O_{21}$	0.46	331.3334	1	959.5297, 779.4575, 633.4018, 471.3490, 391.2820	floralginsenoside H or isomer（$C_{24}H_{40}O_4$-2Glc-Xyl-Dimal）	PGL
**18**	4.43	1023.5376	–H, +HCOO	$C_{48}H_{82}O_{20}$	–0.49	330.1241	1	977.5356, 797.4703, 653.4232, 491.3776	yesanchinoside B or isomer（OT-3Glc）	PGL
**19**	4.56	1255.5926	–H, +HCOO	$C_{57}H_{94}O_{27}$	–3.11	356.5983	1	1209.5893, 1077.5478, 897.4840, 765.4409, 603.3943, 441.3330	notoginsenosides NL-J or isomer（$C_{30}H_{50}O_2$-3Glc-2Xyl-H_2O）	PNL
**20**	4.64	993.5289	–H	$C_{48}H_{82}O_{21}$	1.28	326.8244	1	781.4419, 551.3724, 457.3310	floralginsenoside K or isomer（$C_{30}H_{50}O_3$-GlurA-2Glc-2H_2O）	PGL
**21**	4.65	855.3308	–H	$C_{40}H_{56}O_{20}$	1.89	285.3183	0	837.3175, 761.3056, 718.3017, 529.1489, 491.1671	OT-Rha-Xyl-Mal	PQL
**22**	4.73	1123.5560	–H, +HCOO	$C_{52}H_{86}O_{23}$	1.66	334.7877	0	1077.5480, 945.5038, 765.4449, 603.3872, 441.3361	$C_{30}H_{50}O_2$-3Glc-Xyl-H_2O	PNL
**23**	4.85	1153.6009	–H, +HCOO	$C_{54}H_{92}O_{23}$	–0.18	341.8606	1	1107.5954, 945.5426, 799.4810, 637.4316, 475.3768	ginsenoside Re8 or isomer（PPT-3Glc-Rha）	PGL

**24**	4.91	861.4840	–H, +HCOO	$C_{42}H_{72}O_{15}$	–1.62	293.1058	1	815.4784, 653.4262, 491.3739	24(*R*)-majoroside R1 or isomer（OT-2Glc）	PGL
**25**	5.00	1039.5338	–H, +HCOO	$C_{48}H_{82}O_{21}$	0.67	329.5945	1	993.5234, 813.4615, 795.4594, 633.4015, 471.3468, 391.2830	floralginsenoside K or isomer（$C_{24}H_{40}O_4$-2Glc-Rha-Xyl）	PGL
**26**	5.14	1123.5534	–H, +HCOO	$C_{52}H_{86}O_{23}$	–0.71	329.3828	0	1077.5481, 945.5048, 765.4423, 603.3893, 441.3332	$C_{30}H_{50}O_2$-3Glc-Xyl-H_2O	PNL
**27**	5.21	1255.5963	–H, +HCOO	$C_{57}H_{94}O_{27}$	–0.08	357.9143	1	1209.5894, 1077.5477, 945.5048, 765.4431, 603.3909, 441.3383	notoginsenosides NL-J or isomer（$C_{30}H_{50}O_2$-3Glc-2Xyl-H_2O）	PNL
**28**	5.26	1007.5435	–H, +HCOO	$C_{48}H_{82}O_{19}$	0.28	325.0017	1	961.5373, 799.4838, 637.4322, 475.3792, 391.2828	20-glucoginsenoside-Rf or isomer（PPT-3Glc）	PGL
**29**	5.31	1153.5637	–H, +HCOO	$C_{53}H_{88}O_{24}$	–1.02	335.4078	0	1107.5580, 975.5096, 813.4621, 633.4019, 491.3678	OT-2Glc-2Rha	PNL
**30**	5.35	1123.5874	–H, +HCOO	$C_{53}H_{90}O_{22}$	–2.97	329.9878	1	1077.5854, 945.5437, 783.4907, 637.4314, 475.3787, 391.2835	floralginsenoside M or isomer（PPT-2Glc-Rha-Xyl）	PGL
**31**	5.41	1071.5207	–H	$C_{49}H_{84}O_{25}$	–2.06	312.7639	0	1071.5213, 853.2646, 715.4271, 553.3727, 391.3209	$C_{24}H_{40}O_4$-GlurA-3Glc-H_2O	PNL
**32**[a]	5.43	977.5324	–H, +HCOO	$C_{47}H_{80}O_{18}$	–0.31	310.9244	1	801.3646, 669.5836, 553.3723	notoginsenoside Fp1	PGL、PNL、PQL
**33**	5.46	845.4907	–H, +HCOO	$C_{42}H_{72}O_{14}$	0.35	285.6975	1	751.3661, 675.2654, 591.1338, 391.285	ginsenoside Rg7 or isomer（$C_{24}H_{40}O_4$-2Glc-2Ace）	PQL、PGL
**34**	5.48	989.5337	–H, +HCOO	$C_{48}H_{80}O_{18}$	1.04	321.3499	1	943.5221, 763.4640, 617.4056, 391.2849	ginsenoside Rh18 or isomer（$C_{24}H_{40}O_4$-GlurA-2Rha-2Ace）	PGL
**35**	5.58	831.4756	–H, +HCOO	$C_{41}H_{70}O_{14}$	1	286.6473	1	785.4644, 653.4259, 491.3741	pseudoginsenoside RT2 or isomer（OT-Glc-Xyl）	PGL
**36**[a]	5.61	977.5331	–H, +HCOO	$C_{47}H_{80}O_{18}$	0.41	318.6038	1	475.3786, 391.2882	notoginsenoside R1	PGL、PNL
**37**	5.66	991.5103	–H, +HCOO	$C_{47}H_{78}O_{19}$	–1.68	314.8617	0	945.5051, 765.4485, 603.3901, 441.3353	$C_{30}H_{50}O_2$-3Glc-H_2O	PGL、PNL
**38**	5.67	1123.5894	–H, +HCOO	$C_{53}H_{90}O_{22}$	–1.14	330.4010	1	953.2258, 475.3774	floralginsenoside M or isomer（PPT-2Glc-Rha-Xyl）	PQL、PGL
**39**	5.75	845.4904	–H, +HCOO	$C_{42}H_{72}O_{14}$	–0.04	291.6491	1	799.4869, 653.4260, 491.3734	24(*R*)-pseudoginsenoside F11 or isomer（OT-Glc-Rha）	PGL
**40**	5.83	1167.5812	–H, +HCOO	$C_{54}H_{90}O_{24}$	0.73	344.0055	1	1121.5765, 959.5112, 797.4664, 635.4086, 475.3866	notoginsenoside B or isomer（PPT-GlurA-2Glc-Rha）	PGL
**41**	5.86	977.5334	–H, +HCOO	$C_{47}H_{80}O_{18}$	0.77	313.1896	1	931.5274, 769.4782, 637.4287, 475.3811, 391.2833	ginsenoside Re4 or isomer（PPT-2Glc-Xyl）	PGL
**42**	6.12	1163.5483	–H	$C_{55}H_{88}O_{26}$	–0.71	329.3629	1	1119.5719, 1077.5472, 1059.5417, 945.5064, 765.4460, 603.3909, 441.3343	isomer of m-ginsenoside Rc（$C_{30}H_{50}O_2$-3Glc-Xyl-Mal-3H_2O-CO_2）	PNL
**43**	6.24	835.4628	–H	$C_{48}H_{68}O_{12}$	–1.23	289.6873	0	527.3080, 441.3351	$C_{30}H_{50}O_2$-Glc-Rha-Mal	PGL
**44**[a]	6.25	845.4909	–H, +HCOO	$C_{42}H_{72}O_{14}$	0.59	295.5282	1	529.2974, 457.1596, 255.0293	ginsenoside Rg1	PGL、PNL、PQL
**45**	6.25	917.4211	–H	$C_{40}H_{70}O_{23}$	–2.63	311.5754	0	639.4380, 475.3791	PPT-2Rha-Xyl-H_2O	PGL
**46**	6.25	1025.5541	–H, +HCOO	$C_{48}H_{84}O_{20}$	0.3	332.2994	1	979.5479, 817.4932, 799.4817, 655.4359, 475.3696	ginsengenin-S3 or isomer（PPT-3Glc-2H_2O）	PNL、PQL
**47**[a]	6.35	945.5430	–H	$C_{48}H_{82}O_{18}$	0.18	321.1382	1	475.3792, 391.2856	ginsenoside Re	PGL、PNL、PQL
**48**	6.36	1063.4792	–H	$C_{53}H_{76}O_{22}$	3.47	338.6419	0	1003.4606, 945.5424, 783.4888, 619.4204, 475.3789	PPT-GlueA-2Glc-2CO_2	PQL
**49**	6.46	693.2769	–H	$C_{34}H_{46}O_{15}$	0.69	254.5441	0	475.3789, 457.3688	PPT-GlurA-Ace-H_2O	PGL

**50**	6.56	991.5484	–H, +HCOO	$C_{48}H_{82}O_{18}$	0.16	323.4681	1	945.5427, 783.4921, 637.4324, 475.3789, 391.2829	ginsenoside Rg18 or isomer（PPT-3Glc）	PGL
**51**	6.74	989.5325	–H, +HCOO	$C_{48}H_{80}O_{18}$	–0.18	319.5465	0	943.5298, 781.4739, 635.4157, 475.3757, 391.2826	PPT-GlurA-2Rha	PGL
**52**	6.75	1169.5954	–H, +HCOO	$C_{54}H_{92}O_{24}$	–0.61	350.5709	1	1123.5906, 961.5338, 799.4872, 637.4262, 475.3817	isomer of ginsenoside V（PPT-4Glc）	PGL
**53**	6.76	861.4845	–H, +HCOO	$C_{42}H_{72}O_{15}$	–0.93	288.5399	1	815.4788, 699.3955, 553.3359, 489.3589, 391.2846	ginsenoside Rg12 or isomer（$C_{24}H_{40}O_4$-Glc-Rha-Xyl-2Ace）	PQL、PGL
**54**	6.84	1025.5531	–H, +HCOO	$C_{48}H_{84}O_{20}$	–0.72	333.6497	1	979.5478, 817.4952, 799.4845, 655.4427, 475.3802	ginsengenin-S3 or isomer（PPT-3Glc-2H_2O）	PGL、PNL、PQL
**55**	6.92	991.5476	–H, +HCOO	$C_{48}H_{82}O_{18}$	–0.74	322.5412	1	945.5410, 783.4851, 637.4315, 475.3793, 391.2853	ginsenoside Rg18 or isomer（PPT-3Glc）	PGL
**56**	6.99	845.4917	–H, +HCOO	$C_{42}H_{72}O_{14}$	1.66	298.4877	1	799.4845, 653.4267, 491.3736	24(*R*)-pseudoginsenoside F11 or isomer（OT-Glc-Rha）	PQL
**57**	7.00	699.4329	–H, +HCOO	$C_{36}H_{62}O_{10}$	0.6	271.9149	1	513.1210, 459.2246	ginsenoside M7cd or isomer（$C_{22}H_{36}O_{10}$-GlurA-H_2O）	PGL
**58**	7.05	1271.6278	–H, +HCOO	$C_{58}H_{98}O_{27}$	0.11	369.3899	1	1225.6210, 1093.5774, 913.5153, 781.4747, 619.4211, 537.3355, 375.2898	notoginsenosides NL-B2 or isomer（$C_{24}H_{40}O_3$-GlurA-2Glc-2Xyl-Bute-H_2O）	PNL
**59**	7.07	1031.5438	–H	$C_{51}H_{84}O_{21}$	0.56	326.7446	1	945.5423, 783.4922, 637.4301, 475.3802, 391.2828	malonylfloralginsenoside Re2 or isomer（PPT-2Glc-Rha-Mal）	PGL
**60**	7.20	887.5004	–H, +HCOO	$C_{44}H_{74}O_{15}$	–0.69	303.4521	1	841.4952, 781.4871, 637.4321, 475.3807, 391.2834	notoginsenoside Rt or isomer（PPT-2Glc-Ace）	PGL
**61**	7.31	1169.5959	–H, +HCOO	$C_{54}H_{92}O_{24}$	–0.14	365.2310	1	1123.5881, 961.5142, 781.4737, 619.4224, 537.3445, 375.2941	notoginsenoside A or isomer（$C_{24}H_{40}O_3$-GlurA-3Glc-Bute-H_2O）	PNL
**62**	7.34	1139.5861	–H, +HCOO	$C_{53}H_{90}O_{23}$	0.53	345.6932	1	1093.5805, 961.5375, 799.4851, 637.4316, 475.3798, 391.2830	floralginsenoside P or isomer（PPT-3Glc-Xyl）	PGL
**63**	7.38	1033.5573	–H, +HCOO	$C_{50}H_{84}O_{19}$	–1.65	330.3589	1	621.4049, 457.3702, 391.2848	6'''-*O*-acetylginsenoside Re or isomer（PPT-2Glc-Rha-3Ace）	PGL
**64**	7.39	1271.6273	–H, +HCOO	$C_{58}H_{98}O_{27}$	–0.35	385.1713	1	1225.6230, 1093.5819, 913.5162, 781.4741, 619.4214, 537.3435, 375.3029	notoginsenosides NL-B2 or isomer（$C_{24}H_{40}O_3$-GlurA-2Glc-2Xyl-Bute-H_2O）	PNL
**65**	7.49	1323.6601	–H	$C_{62}H_{102}O_{27}$	0.83	365.4381	0	1013.5337, 945.5419, 783.4900, 637.4333, 475.3852, 391.2865	PPT-2Glc-2GlurA-Dimal	PGL
**66**	7.50	1065.5492	–H, +HCOO	$C_{50}H_{84}O_{21}$	0.49	338.9945	1	979.5452, 799.4841, 655.4398, 491.8954	floralginsenoside H or isomer（OT-3Glc-2CO_2）	PGL
**67**	7.52	959.5225	–H	$C_{48}H_{80}O_{19}$	0.46	327.9616	1	687.3303, 457.3281, 391.2854	vinaginsenoside R20 or isomer（$C_{24}H_{40}O_4$-GlurA-Glc-Rha-2Ace）	PQL、PGL
**68**	7.57	1031.5445	–H	$C_{51}H_{84}O_{21}$	1.27	324.5273	1	945.5437, 783.4865, 637.4320, 475.3791, 391.2857	malonylfloralginsenoside Re2 or isomer（PPT-2Glc-Rha-Mal）	PQL、PGL
**69**	7.65	1139.5847	–H, +HCOO	$C_{53}H_{90}O_{23}$	–0.71	354.1549	1	1093.5785, 781.4732, 619.4229, 375.2838	floranotoginsenoside A or isomer（$C_{24}H_{40}O_3$-GlurA-2Glc-Xyl-Mal）	PGL、PNL、PQL
**70**	7.72	847.4704	–H, +HCOO	$C_{41}H_{70}O_{15}$	0.82	292.5686	1	517.2589, 375.1673	floralginsenoside C or isomer（$C_{24}H_{40}O_3$-Glc-2Xyl）	PGL
**71**	7.74	1007.5443	–H, +HCOO	$C_{48}H_{82}O_{19}$	1.15	330.3229	1	961.5393, 799.4858, 637.4317, 475.3797, 391.2840	20-glucoginsenoside-Rf or isomer（PPT-3Glc）	PGL
**72**	7.78	1271.6258	–H, +HCOO	$C_{58}H_{98}O_{27}$	–1.54	365.7701	1	1225.6199, 1093.5779, 961.5310, 799.4845, 781.4729, 637.4304, 475.3764	isomer of chikusetsusaponin LM6（PPT-3Glc-2Xyl）	PNL

**73**	7.79	1169.5928	–H, +HCOO	$C_{54}H_{92}O_{24}$	–2.94	347.5191	1	1123.5886, 961.5256, 799.4874, 781.4679, 637.4332, 475.3799	isomer of notoginsenoside A（PPT-4Glc）	PNL
**74**	7.80	1139.5861	–H, +HCOO	$C_{53}H_{90}O_{23}$	0.58	358.5364	1	1093.5808, 961.5383, 799.4837, 637.4314, 475.3784, 391.2838	floralginsenoside P or isomer（PPT-3Glc-Xyl）	PGL
**75**	7.82	653.4277	–H	$C_{36}H_{62}O_{10}$	1.06	270.5793	1	459.3480, 373.2743	ginsenoside M7cd or isomer（$C_{30}H_{52}O_3$-GlurA-H_2O）	PGL
**76**	7.83	961.5368	–H, +HCOO	$C_{47}H_{80}O_{17}$	–1.06	314.1075	1	915.5291, 753.4819, 607.4190, 589.4054, 475.3792, 391.2845	quinquenoside L14 or isomer（PPT-Glc-Rha-Xyl）	PQL
**77**	7.88	815.4802	–H	$C_{42}H_{72}O_{15}$	0.52	288.7003	1	639.4390, 475.3790, 349.2739	ginsenoside Rg12 or isomer（$C_{30}H_{52}O_4$-GlurA-Rha-H_2O）	PGL
**78**	7.92	1033.5580	–H, +HCOO	$C_{50}H_{84}O_{19}$	–0.93	334.3474	1	945.5429, 783.4896, 637.4326, 475.3794, 391.2854	pseudoginsenoside RC1 or isomer（PPT-2Glc-Rha-Ace）	PGL
**79**[a]	7.93	1031.5438	–H	$C_{51}H_{84}O_{21}$	0.55	319.9784	1	921.2063, 643.1047, 441.1226	malonylfloralginsenoside Re1	PGL、PNL
**80**	7.94	827.4805	–H	$C_{43}H_{72}O_{15}$	0.83	292.8315	1	783.4896, 637.4322, 619.4211, 475.3790	PPT-Glc-Rha-CO_2	PGL
**81**	7.95	841.4957	–H	$C_{44}H_{74}O_{15}$	0.29	295.0329	1	799.4856, 637.4322, 475.3790	notoginsenoside Rt or isomer（PPT-2Glc-Ace）	PGL
**82**	8.02	1139.5861	–H, +HCOO	$C_{53}H_{90}O_{23}$	0.51	357.8098	1	1093.5798, 961.5369, 781.4744, 619.4211, 475.3834	floralginsenoside P or isomer（PPT-3Glc-Xyl）	PNL、PQL
**83**	8.06	935.4855	–H	$C_{45}H_{76}O_{20}$	–0.16	306.3408	1	743.2231, 615.3951, 459.2202	floralginsenoside Kc or isomer（$C_{22}H_{36}O_{10}$-Glc-Rha-Xyl-2H_2O）	PGL
**84**	8.14	1185.5914	–H, +HCOO	$C_{54}H_{92}O_{25}$	0.38	363.1953	1	1139.5850, 1121.5768, 797.4671, 765.4439, 603.3947, 441.3436	notoginsenoside K or isomer（$C_{30}H_{50}O_2$-GlurA-3Glc-2H_2O）	PGL、PNL
**85**	8.16	961.5392	–H, +HCOO	$C_{47}H_{80}O_{17}$	1.53	320.7601	1	915.5345, 783.4948, 637.4335, 475.3781, 391.2798	ginsenoside Rh24 or isomer（PPT-Glc-Rha-Xyl）	PGL
**86**	8.22	1033.5581	–H	$C_{50}H_{84}O_{19}$	–0.85	325.3973	1	945.5425, 783.4955, 637.4314, 475.3786, 391.2835	6'''-*O*-acetylginsenoside Re or isomer（PPT-2Glc-Rha-Ace）	PGL
**87**	8.23	1241.6164	–H	$C_{58}H_{98}O_{28}$	–0.65	382.5807	0	765.4470, 603.3895, 441.3375	isomer of notoginsenosides NL-A3（$C_{30}H_{50}O_2$-3Glc-Rha-Xyl-2H_2O）	PNL
**88**	8.27	1225.6226	–H	$C_{58}H_{98}O_{27}$	0.26	373.7117	1	985.4420, 835.3929, 765.4470, 603.3895, 475.3782	isomer of chikusetsusaponin LM6（PPT-3Glc-2Xyl）	PNL
**89**[a]	8.37	815.4818	–H, +HCOO	$C_{41}H_{70}O_{13}$	2.64	293.1066	1	619.4251, 475.3802, 353.2725	20(*S*)-sanchirhinoside A3	PQL、PGL
**90**	8.40	961.5400	–H, +HCOO	$C_{47}H_{80}O_{17}$	2.4	320.3556	1	915.5340, 783.4926, 637.4322, 475.3802, 391.2853	ginsenoside Rh24 or isomer（PPT-Glc-Rha-Xyl）	PGL
**91**	8.43	1139.5858	–H, +HCOO	$C_{53}H_{90}O_{23}$	0.27	352.1670	1	1093.5804, 961.5397, 799.4846, 781.4745, 637.4311, 475.3790	floralginsenoside P or isomer（PPT-3Glc-Xyl）	PGL、PNL、PQL
**92**	8.44	993.5293	–H, +HCOO	$C_{47}H_{80}O_{19}$	1.76	325.1149	1	947.5202, 785.4682, 623.4181, 461.3655, 475.3815	notoginsenoside H or isomer（PPT-3Glc-H_2O）	PQL、PGL
**93**	8.50	1109.5730	–H	$C_{53}H_{90}O_{24}$	–1.74	347.1267	1	1109.5797, 945.5598, 765.4462, 459.3878	floranotoginsenoside B or isomer（PPD-3Glc-Rha-H_2O）	PGL、PNL
**94**	8.53	989.5341	–H, +HCOO	$C_{48}H_{80}O_{18}$	1.45	322.3253	1	943.5291, 797.4709, 635.4132, 391.2832	ginsenoside Rh18 or isomer（$C_{24}H_{40}O_4$-GlurA-2Rha-2Ace）	PGL
**95**	8.58	1269.6100	–H, +HCOO	$C_{58}H_{96}O_{27}$	–1.69	366.9162	1	1223.6085, 1091.5664, 929.5181, 779.4585, 635.4104, 455.3468	notoginsenoside LK4 or isomer（$C_{30}H_{48}O_3$-2Glc-Rha-GlurA-2H_2O）	PNL
**96**[a]	8.62	961.5376	–H	$C_{48}H_{82}O_{19}$	–0.09	326.8923	1	799.4858, 537.3431, 453.3551, 373.2743	vinaginsenoside R8	PGL、PNL、PQL

**97**	8.75	1139.5844	–H	$C_{54}H_{92}O_{25}$	–0.99	358.7544	1	945.5424, 765.4452, 441.3355	notoginsenoside K or isomer（$C_{30}H_{50}O_2$-GlurA-3Glc-2H_2O）	PGL
**98**	8.78	1117.5461	–H	$C_{54}H_{86}O_{24}$	2.24	321.7158	0	999.5222, 797.4686, 765.4453, 453.3353	malonylfloralginsenoside Rd6 or isomer（$C_{30}H_{45}O_3$-2Glc-Xyl-Dimal-2H_2O）	PGL
**99**[a]	8.84	961.5382	–H	$C_{48}H_{82}O_{19}$	0.53	325.9221	1	723.3535, 621.4079, 475.3685, 391.2845	vinaginsenoside R4	PQL、PGL
**100**	8.89	1155.5824	–H, +HCOO	$C_{53}H_{90}O_{24}$	1.8	365.9944	1	1109.5758, 959.5160, 797.4699, 765.4434, 603.3875, 441.3372	floranotoginsenoside B or isomer（$C_{30}H_{50}O_2$-GlurA-2Glc-Xyl-2H_2O）	PGL、PNL、PQL
**101**	8.92	871.4698	–H	$C_{44}H_{72}O_{17}$	0.19	287.3434	1	765.4442, 637.4355, 441.3044	notoginsenosides NL-A4 or isomer（$C_{30}H_{50}O_2$-Glc-Rha-Mal-2H_2O）	PNL
**102**	8.94	977.5337	–H	$C_{48}H_{82}O_{20}$	1.17	320.1125	1	851.4015, 765.4387, 605.3052, 375.2892	ginsenoside I or isomer（$C_{24}H_{40}O_3$-GlurA-2Glc-2Ace-H_2O）	PGL
**103**	8.95	1139.5868	–H, +HCOO	$C_{53}H_{90}O_{23}$	1.2	358.1407	1	1093.5810, 961.5382, 799.4852, 781.4837, 637.4307, 475.3781	floralginsenoside P or isomer（PPT-3Glc-Xyl）	PGL、PNL、PQL
**104**	8.98	1241.6148	–H	$C_{58}H_{98}O_{28}$	–1.9	368.1181	1	1093.5800, 767.1622, 375.2895	notoginsenosides NL-A_3（$C_{24}H_{40}O_3$-GlurA-2Glc-2Xyl-2Ace-H_2O）	PNL
**105**	9.05	1179.5808	–H	$C_{56}H_{92}O_{26}$	0.34	349.2273	0	779.4504, 617.4028, 455.3537	OA-GlurA-3Glc-CO_2-H_2O	PNL、PQL
**106**	9.08	1007.5437	–H, +HCOO	$C_{48}H_{82}O_{19}$	0.53	326.5110	1	961.5388, 799.4850, 781.4771, 637.4313, 475.3796, 391.2812	20-glucoginsenoside-Rf or isomer（PPT-3Glc）	PGL、PNL、PQL
**107**	9.09	1269.6141	–H, +HCOO	$C_{58}H_{96}O_{27}$	1.66	365.3296	1	1223.6056, 1091.5644, 959.5195, 797.4689, 779.4584, 617.4051, 455.3579	notoginsenoside LK4 or isomer（$C_{30}H_{48}O_3$-2Glc-Rha-GlurA-2H_2O）	PNL
**108**	9.10	1109.5740	–H	$C_{53}H_{90}O_{24}$	–0.77	347.4275	1	961.5418, 757.2215, 455.3508	floranotoginsenoside B or isomer（$C_{30}H_{48}O_3$-3Glc-Xyl-2H_2O）	PNL、PQL
**109**	9.16	1137.5700	–H, +HCOO	$C_{53}H_{88}O_{23}$	0.14	338.0425	1	1091.5634, 959.5247, 779.4591, 617.4014, 375.2901	Notoginsenoside Ng2 or isomer（$C_{24}H_{40}O_3$-GlurA-2Glc-Xyl-2Ace）	PGL、PNL
**110**	9.25	1047.5385	–H	$C_{51}H_{84}O_{22}$	0.36	338.6342	0	1003.5481, 961.5359, 783.4812, 619.4221, 537.3435, 375.2932	$C_{24}H_{40}O_3$-2Glc-2Xyl-2Ace	PNL、PQL
**111**	9.34	1195.5754	–H	$C_{56}H_{92}O_{27}$	0.05	362.6939	0	1091.5566, 959.5360, 839.4789, 797.4714, 765.4433, 635.4196, 375.2873	$C_{24}H_{40}O_3$-2Glc-Rha-Xyl-GlurA-Ace	PGL
**112**	9.43	1007.5437	–H, +HCOO	$C_{48}H_{82}O_{19}$	0.48	328.6909	1	961.5390, 799.4854, 781.4739, 637.4309, 475.3790	20-glucoginsenoside-Rf or isomer（PPT-3Glc）	PGL、PNL、PQL
**113**	9.44	831.4750	–H, +HCOO	$C_{41}H_{70}O_{14}$	0.24	293.0606	1	785.4680, 653.4273, 491.3726	pseudoginsenoside RT2 or isomer（OT-Glc-Xyl）	PQL
**114**	9.47	1033.5586	–H, +HCOO	$C_{50}H_{84}O_{19}$	–0.29	332.4950	1	945.5405, 799.4879, 637.4318, 475.3785, 391.2854	6'''-*O*-acetylginsenoside Re or isomer（PPT-2Glc-Rha-Ace）	PGL
**115**	9.48	1155.5805	–H, +HCOO	$C_{53}H_{90}O_{24}$	0.11	357.3715	1	1109.5701, 959.5115, 797.4671, 779.4592, 617.4053, 455.3515	floranotoginsenoside B or isomer（$C_{30}H_{48}O_3$-3Glc-Xyl-2H_2O）	PGL、PNL
**116**	9.50	1023.5385	–H, +HCOO	$C_{48}H_{82}O_{20}$	0.42	322.4410	1	977.5311, 797.4684, 765.4419, 603.3886, 375.2912	ginsenoside I or isomer（$C_{24}H_{40}O_3$-2Glc-Rha-Xyl）	PQL、PGL
**117**	9.65	1109.5719	–H	$C_{53}H_{90}O_{24}$	–2.71	347.8903	1	853.4063, 799.4775, 537.3454, 375.2894	floranotoginsenoside B or isomer（$C_{24}H_{40}O_3$-GlurA-Glc-2Rha-Mal-H_2O）	PGL、PNL
**118**	9.65	1047.5398	–H	$C_{51}H_{84}O_{22}$	1.61	339.2090	0	1003.5554, 961.5376, 823.4964, 781.4742, 637.4312, 475.3790	PPT-3Glc-Mal	PQL
**119**	9.70	1137.5692	–H, +HCOO	$C_{53}H_{88}O_{23}$	–0.55	349.2483	1	1091.5642, 959.5222, 797.4685, 779.4569, 635.4166, 617.4045, 375.2964	Notoginsenoside Ng2 or isomer（$C_{24}H_{40}O_3$-GlurA-2Glc-Xyl-2Ace）	PGL、PNL、PQL

**120**[a]	9.78	1371.6804	–H	$C_{64}H_{108}O_{31}$	0.19	393.8447	1	1239.6297, 945.5430, 783.4909, 621.4372	notoginsenoside T	PNL
**121**[a]	9.80	653.4279	–H	$C_{36}H_{62}O_{10}$	1.33	270.8075	1	349.2518, 329.1897, 217.1589	pseudoginsenoside Rt5 (24*R*)	PQL
**122**[a]	9.81	799.4856	–H	$C_{42}H_{72}O_{14}$	0.86	298.2295	1	707.3480, 635.4158, 491.3736	24(*R*)-pseudoginsenoside F11	PQL、PGL
**123**	9.88	1005.5287	–H, +HCOO	$C_{48}H_{80}O_{19}$	1.12	326.1424	1	959.5221, 797.4687, 779.4589, 617.4049, 455.3542	notoginsenoside G or isomer（$C_{30}H_{48}O_3$-3Glc-H_2O）	PGL
**124**	9.92	975.5533	–H, +HCOO	$C_{48}H_{82}O_{17}$	–0.06	317.8858	1	929.5475, 767.4953, 621.4367, 475.3791, 391.2847	vinaginsenoside R3 or isomer（PPT-Glc-2Rha）	PGL
**125**	10.05	991.5123	–H	$C_{48}H_{80}O_{21}$	0.36	316.5524	0	879.3737, 765.4410, 609.2709, 375.2896	$C_{24}H_{40}O_3$-2Glc-2Rha	PQL
**126**	10.06	1023.5382	–H, +HCOO	$C_{48}H_{82}O_{20}$	0.05	328.7434	1	879.3737, 765.4410, 609.2709, 547.0343, 375.2896	ginsenoside I or isomer（$C_{24}H_{40}O_3$-GlurA-2Glc-2Ace-H_2O）	PQL、PGL
**127**	10.08	1047.5391	–H	$C_{51}H_{84}O_{22}$	0.91	329.9378	0	1003.5551, 961.5373, 943.5271, 799.4865, 781.4734, 637.4314, 619.4305, 475.3867, 391.2897	PPT-3Glc-Ace	PGL、PNL、PQL
**128**	10.10	1049.5538	–H, +HCOO	$C_{50}H_{84}O_{20}$	0.02	338.5780	1	1003.5459, 961.5381, 943.5270, 799.4844, 781.4721, 637.4336, 475.3783, 391.2822	6-acetyl ginsenoside Rg3 or isomer（PPT-3Glc-Ace）	PGL
**129**	10.13	1195.5748	–H	$C_{56}H_{92}O_{27}$	–0.39	344.2513	0	1151.6013, 1109.5757, 1091.5632, 959.5480, 797.4705, 779.4595, 635.4170, 617.4098, 455.3522	OA-Glc-Rha-3Xyl-2H_2O	PNL
**130**	10.17	801.4646	–H	$C_{41}H_{70}O_{15}$	0.46	292.9902	1	741.4646, 535.0825, 375.2892	floralginsenoside C or isomer（$C_{24}H_{40}O_3$-Glc-2Xyl）	PGL、PNL、PQL
**131**	10.20	1063.5343	–H	$C_{51}H_{84}O_{23}$	1.24	339.0396	0	1019.5504, 797.4679, 765.4432, 635.4145, 617.4061, 455.3519	OA-2Glc-Xyl-2Ace-Bute	PQL、PGL
**132**	10.37	1139.5849	–H, +HCOO	$C_{53}H_{90}O_{23}$	–0.55	354.8594	1	1093.5798, 931.5271, 799.4823, 637.4347, 475.3763,	floralginsenoside P or isomer（PPT-3Glc-Xyl）	PGL
**133**	10.38	959.5217	–H	$C_{48}H_{80}O_{19}$	–0.4	323.9329	1	959.5223, 797.4728, 635.4147, 599.3975, 473.3623, 455.3524	notoginsenoside G or isomer（$C_{30}H_{48}O_3$-3Glc-H_2O）	PGL、PNL、PQL
**134**	10.39	845.4899	–H, +HCOO	$C_{42}H_{72}O_{14}$	–0.59	295.8028	1	621.2330, 375.2896	notoginsenoside U or isomer（$C_{24}H_{40}O_3$-Glc-Xyl-Bute-CO_2-H_2O)	PNL
**135**	10.39	991.5133	–H	$C_{48}H_{80}O_{21}$	1.43	316.4481	0	613.3156, 599.4000, 437.3416, 375.2886	$C_{24}H_{40}O_3$-2Glc-2Rha	PQL
**136**	10.41	1341.6685	–H	$C_{63}H_{106}O_{30}$	–0.8	379.6677	1	1077.5898, 783.4893, 621.4377, 537.3461, 443.3483	notoginsenoside Q or isomer（$C_{30}H_{52}O_2$-2Glc-3Xyl-2Bute-Ace）	PNL
**137**	10.47	1269.6469	–H	$C_{60}H_{102}O_{28}$	–1.19	372.0802	1	783.4893, 621.4377, 459.3820, 375.2896	ginsenoside Rb5 or isomer（PPD-5Glc）	PNL
**138**	10.54	1063.5324	–H	$C_{51}H_{84}O_{23}$	–0.53	342.0060	0	979.5413, 797.4685, 617.4009, 455.3535	OA-2Glc-Xyl-2Ace-Bute	PGL
**139**[a]	10.57	845.4911	–H, +HCOO	$C_{42}H_{72}O_{14}$	0.82	302.0425	1	799.4838, 637.4315, 475.3793, 391.2841	ginsenoside Rf	PGL
**140**[a]	10.68	1239.6383	–H	$C_{59}H_{100}O_{27}$	0.36	365.9795	1	1077.5813, 897.5236, 783.4910, 621.4365, 459.3843, 375.2904	notoginsenoside Fa	PNL
**141**	10.68	977.5328	–H, +HCOO	$C_{47}H_{80}O_{18}$	0.12	321.3157	1	931.5283, 799.4854, 637.4312, 475.3791, 391.2840	notoginsenoside FP1 or isomer(PPT-2Glc-Xyl）	PGL、PNL
**142**	10.78	1065.5483	–H, +HCOO	$C_{50}H_{84}O_{21}$	–0.35	341.2495	1	977.5434, 797.4700, 765.4456, 641.3523, 457.3403	floralginsenoside H or isomer（$C_{30}H_{50}O_3$-GlurA-Glc-Rha-Ace-2H_2O）	PGL

**143**	10.86	1151.5858	–H, +HCOO	$C_{54}H_{90}O_{23}$	0.25	347.3729	1	1105.5815, 943.5269, 781.4742, 619.4195, 457.3708	5,6-didehydroginsenoside Rb1 or isomer（$C_{30}H_{50}O_{3}$-4Glc）	PGL
**144**	10.90	1063.5331	–H	$C_{51}H_{84}O_{23}$	0.05	336.4027	0	979.5428, 797.4729, 779.4651, 617.3992, 455.3622	OA-2Glc-Xyl-2Ace-Bute	PGL
**145**	10.93	989.5332	–H, +HCOO	$C_{48}H_{80}O_{18}$	0.54	328.2912	1	943.5269, 781.4713, 619.4227, 457.3789	ginsenoside Rh18 or isomer（$C_{30}H_{50}O_{3}$-3Glc）	PGL
**146**	10.93	1285.6437	–H, +HCOO	$C_{59}H_{100}O_{27}$	0.2	369.2964	1	1239.6364, 1077.5845, 945.5410, 783.4897, 621.4349, 459.3842, 375.2975	ginsenoside Ra3 or isomer（PPD-4Glc-Xyl）	PNL
**147**	11.06	1387.6744	–H, +HCOO	$C_{63}H_{106}O_{30}$	–0.5	380.5737	1	1341.6690, 1209.6262, 1077.5840, 945.5443, 783.4892, 621.4383, 459.3826	notoginsenoside Q or isomer（PPD-2Glc-Rha-Xyl-GlurA-Bute-2H_2O）	PNL
**148[a]**	11.11	1209.6276	–H	$C_{58}H_{98}O_{26}$	0.25	357.9905	1	999.4559, 879.4133, 747.3764, 537.3441, 459.3843	ginsenoside Ra2	PNL
**149[a]**	11.12	815.4820	–H, +HCOO	$C_{41}H_{70}O_{13}$	2.87	300.1277	1	815.4803, 783.4903, 621.4373, 475.3794, 391.2856	ginsenoside F5	PGL、PNL、PQL
**150**	11.16	1325.6377	–H	$C_{62}H_{102}O_{30}$	–0.46	369.9505	1	1281.6504, 1107.5929, 1089.5905, 1077.5793, 945.5450, 783.4881, 621.4407, 459.3966	malonylginsenoside Ra3 or isomer（PPD-4Glc-Xyl-Mal）	PNL
**151**	11.20	1107.5964	–H	$C_{54}H_{92}O_{23}$	0.69	270.9253	1	621.4379, 459.3837, 375.2890	ginsenoside Rb1 or isomer（PPD-4Glc）	PGL、PNL、PQL
**152**	11.21	977.5307	–H	$C_{48}H_{82}O_{20}$	–1.92	320.6493	1	945.5540, 783.4882, 621.4366, 491.3645	yesanchinoside B or isomer（OT-3Glc）	PNL、PQL
**153**	11.30	1121.5757	–H, +HCOO	$C_{53}H_{88}O_{22}$	0.72	340.2801	1	1075.5797, 913.5180, 781.4794, 457.3754	quinquefoloside-Lb or isomer（$C_{30}H_{50}O_{3}$-3Glc-Xyl）	PGL
**154[a]**	11.32	815.4774	–H, +HCOO	$C_{41}H_{70}O_{13}$	–3.17	298.9475	1	815.4795, 769.4742, 529.2983, 475.3785, 391.2851	ginsenoside F3	PGL、PNL、PQL
**155**	11.33	807.4526	–H	$C_{43}H_{68}O_{14}$	–1.2	294.3666	1	659.2731, 609.1409, 459.3793, 373.2746	isomer of ginsenoside Rs4（PPD-2Rha-H_2O）	PGL
**156**	11.41	1285.6433	–H, +HCOO	$C_{59}H_{100}O_{27}$	–0.08	374.4055	1	945.5424, 769.2605, 373.3166	ginsenoside Ra3 or isomer（5,6-didehydro PPD-GlurA-2Glc-2Xyl-2Ace-H_2O）	PNL
**157**	11.55	807.4529	–H	$C_{43}H_{68}O_{14}$	–0.84	293.3435	1	589.3176, 475.3793, 391.2838	isomer of ginsenoside Rs4（PPT-2Xyl-Bute）	PGL
**158**	11.55	815.4815	–H, +HCOO	$C_{41}H_{70}O_{13}$	2.17	301.3002	1	475.3793, 373.2735	isomer of ginsenoside F5（PPT-Glc-Xyl）	PGL
**159[a]**	11.65	1193.5969	–H	$C_{57}H_{94}O_{26}$	0.79	346.6930	1	1001.4604, 839.4078, 715.3563, 675.3686, 597.3260	m-ginsenoside Rb1	PGL、PNL、PQL
**160[a]**	11.66	1209.6267	–H	$C_{58}H_{98}O_{26}$	–0.48	366.2556	1	1001.4668, 605.4039, 459.3834	ginsenoside Ra1	PNL
**161**	11.68	1195.6101	–H, +HCOO	$C_{56}H_{94}O_{24}$	–1.36	367.2338	1	947.5473, 621.4358, 459.3927, 375.2857	6"-*O*-acetylginsenoside Rb1 or isomer（PPD-4Glc-Ace）	PGL、PNL、PQL
**162**	11.69	1121.5746	–H	$C_{54}H_{90}O_{24}$	–0.24	342.5452	1	999.4573, 675.3686, 441.3739	notoginsenoside B or isomer（$C_{30}H_{50}O_{2}$-GlurA-3Glc-H_2O）	PGL
**163**	11.70	1145.6080	–H	$C_{57}H_{94}O_{23}$	–2.87	271.8239	1	765.4796, 537.3430, 459.3834, 375.2893	isomer of ginsenoside Ra7（PPD-3Glc-Xyl-Bute）	PNL
**164[a]**	11.73	1123.5897	–H, +HCOO	$C_{53}H_{90}O_{22}$	–0.86	343.5379	1	999.4435, 789.2835, 605.4022, 537.3439, 459.3833	ginsenoside Rc	PGL、PNL、PQL
**165**	11.74	1073.5545	–H	$C_{53}H_{86}O_{22}$	0.63	335.1005	0	1073.5491, 765.4793, 721.2731, 591.2502, 459.3833,	PPD-GlurA-2Xyl-Mal-2CO_2	PQL
**166[a]**	11.80	1341.6705	–H	$C_{63}H_{106}O_{30}$	0.69	384.0701	1	1053.7338, 837.4131, 765.4793, 375.2887	notoginsenoside S	PNL

**167**	11.86	1295.6273	–H	$C_{61}H_{100}O_{29}$	–0.33	361.8820	0	1249.5092, 1191.6154, 1077.5823, 1059.5749, 915.5416, 783.4918, 621.4334.459.3798, 375.2877	PPD-Glc-Rha-3Xyl-Mal	PNL
**168**	11.87	1255.6323	–H	$C_{59}H_{100}O_{28}$	–0.42	378.8360	1	1077.5955, 1089.5850, 927.5235, 707.3694, 457.3792	3-*O*-*β*-D-xylopyranosyl-(1→2)-*β*-D-glucopyranosyl-(1→2)-*β*-D-glucopyranosyl-3*β*,12*β*,20*S*,24*S*-tetrahydroxydammar-25-ene-20-*O*-*β*-D-glucopyranosyl-(1→6)-*β*-D-glucopyranoside or isomer（$C_{30}H_{50}O_3$-4Glc-Xyl-H_2O）	PNL
**169**[a]	11.87	829.4962	–H, +HCOO	$C_{42}H_{72}O_{13}$	0.87	298.7653	1	785.4961, 589.3162, 475.3787, 391.2846	ginsenoside Rg2	PGL、PNL、PQL
**170**	11.89	1193.5962	–H	$C_{57}H_{94}O_{26}$	0.17	337.2908	1	1107.5955, 1089.5825, 945.5434, 783.4907, 621.4345, 459.3803	isomer of m-ginsenoside Rb1（PPD-4Glc-Mal）	PQL
**171**	11.91	1121.5753	–H, +HCOO	$C_{53}H_{88}O_{22}$	0.34	349.5755	1	847.4002, 783.4905, 741.5009, 551.3267, 475.3787	quinquefoloside-Lb or isomer（PPT-2GlurA-Glc-Bute-H_2O）	PQL、PGL
**172**	11.92	845.4913	–H, +HCOO	$C_{42}H_{72}O_{14}$	1.08	304.5754	1	783.4905, 581.0785, 475.3787	ginsenoside-La or isomer（PPT-Xyl-Bute-2CO_2-2H_2O)	PQL
**173**[a]	11.95	683.4381	–H, +HCOO	$C_{36}H_{62}O_9$	0.76	275.9583	1	597.6910, 489.1295, 213.1271	ginsenoside Rh1	PGL
**174**	12.03	991.5477	–H, +HCOO	$C_{48}H_{82}O_{18}$	–0.67	328.2080	1	945.5425, 781.4697, 637.4329, 475.3780, 391.2922	ginsenoside Rg18 or isomer（PPT-3Glc）	PGL
**175**	12.08	1255.6325	–H, +HCOO	$C_{58}H_{98}O_{26}$	–0.25	380.7280	1	1209.6262, 1077.5826, 915.5312, 753.4793, 621.4381, 459.3813	notoginsenoside-FZ or isomer（PPD-3Glc-2Xyl）	PNL
**176**	12.14	829.4953	–H, +HCOO	$C_{42}H_{72}O_{13}$	–0.2	304.2829	1	783.4833, 637.4359, 475.3789, 391.2841	isomer of ginsenoside Rg2（PPT-Glc-Rha）	PQL、PGL
**177**	12.26	1101.5885	–H	$C_{55}H_{90}O_{22}$	3.06	262.1909	0	951.3470, 819.3988, 783.4895, 621.3561, 459.3836, 375.2898	PPD-3Glc-Bute-2CO_2	PQL
**178**[a]	12.27	1123.5900	–H, +HCOO	$C_{53}H_{90}O_{22}$	–0.52	356.3329	1	785.4749, 707.3664, 605.4039, 459.3836	ginsenoside Rb2	PGL、PNL、PQL
**179**	12.36	1163.5855	–H	$C_{56}H_{92}O_{25}$	–0.01	344.2092	1	1119.5961, 945.5440, 783.4895, 621.4376, 459.3840	isomer of m-ginsenoside Rc（PPD-3Glc-Xyl-Mal）	PGL、PNL、PQL
**180**	12.39	1295.6276	–H	$C_{61}H_{100}O_{29}$	–0.08	364.5308	0	1251.6401, 1209.6271, 1077.5846, 1059.5718, 945.5466, 915.5313, 783.4852, 621.4429, 459.3830, 375.2928	PPD-2Glc-2Rha-Xyl-2CO_2	PNL
**181**	12.42	815.4800	–H, +HCOO	$C_{41}H_{70}O_{13}$	0.3	297.5575	1	693.3675, 595.2579, 441.0894	notoginsenoside R2 or isomer（$C_{30}H_{50}O_2$-2Rha-2H_2O）	PGL
**182**[a]	12.55	1123.5911	–H, +HCOO	$C_{53}H_{90}O_{22}$	0.49	353.4245	1	953.4847, 839.4144, 717.3663, 605.4140, 459.3846	ginsenoside Rb3	PGL、PNL、PQL
**183**	12.56	799.4829	–H	$C_{42}H_{72}O_{14}$	–2.44	300.6810	1	637.4368, 475.3800, 391.2903	ginsenoside-La or isomer（PPT-2Glc）	PGL
**184**	12.58	1195.5218	–H	$C_{58}H_{84}O_{26}$	3.33	341.8116	0	1109.6206, 1059.5767, 675.3562, 459.3847, 375.2900	PPD-3Glc-Rha-Mal-H_2O	PNL、PQL
**185**	12.59	829.4972	–H, +HCOO	$C_{42}H_{72}O_{13}$	2.13	301.6790	1	691.4311, 615.3083, 441.3761	notoginsenoside SY3 or isomer（$C_{30}H_{50}O_2$-2Glc-H_2O）	PNL
**186**[a]	12.60	635.4149	–H	$C_{36}H_{60}O_9$	–2.4	271.7059	1	525.2773, 443.1931, 329.2538	ginsenoside Rh7	PGL
**187**	12.68	1091.5634	–H	$C_{53}H_{88}O_{23}$	–0.87	332.1887	1	993.3333, 693.3635, 457.3628	Notoginsenoside Ng2 or isomer（$C_{30}H_{50}O_3$-GlurA-Glc-Rha-Xyl-H_2O）	PQL
**188**	12.70	1255.6312	–H, +HCOO	$C_{58}H_{98}O_{26}$	–1.32	364.5043	1	1209.6257, 1077.5860, 1047.5771, 945.5434, 783.4893, 621.4321, 459.3840	notoginsenoside-FZ or isomer（PPD-3Glc-2Xyl）	PNL

**189**	12.71	1163.5844	–H	$C_{56}H_{92}O_{25}$	–0.94	345.4921	1	1119.5946, 1077.5840, 1059.5739, 945.5412, 825.5006, 783.4891, 765.4787, 621.4362, 459.3824, 375.2908	isomer of m-ginsenoside Rc（PPD-3Glc-Xyl-Mal）	PGL、PNL
**190**	12.74	885.4848	–H	$C_{45}H_{74}O_{17}$	–0.59	288.9251	0	841.5006, 799.4885, 517.3896, 375.2817	PPD-Glc-GlurA-Dimal	PNL
**191**	12.74	1193.5957	–H	$C_{57}H_{94}O_{26}$	–0.26	362.1214	1	1149.6066, 1107.5953, 1089.5835, 945.5423, 783.4902, 621.4381, 459.3854, 375.2874	isomer of m-ginsenoside Rb1（PPD-4Glc-Mal）	PQL、PGL
**192**	12.81	1123.5900	–H, +HCOO	$C_{53}H_{90}O_{22}$	–0.56	351.5175	1	1077.5849, 945.5420, 783.4903, 621.4365, 459.3842, 375.2911	notoginsenoside L or isomer（PPD-3Glc-Xyl）	PNL
**193**	12.84	1165.5996	–H, +HCOO	$C_{55}H_{92}O_{23}$	–1.36	366.1348	1	1119.6037, 1077.5850, 1059.5798, 945.5378, 783.4897, 765.4828, 621.4400, 459.3857	pseudoginsenoside F8 or isomer（PPD-3Glc-Xyl-Ace）	PNL
**194**[a]	12.84	955.4908	–H	$C_{48}H_{76}O_{19}$	0.07	320.6009	1	955.4960, 571.4021, 465.1603	ginsenoside Ro	PGL、PNL、PQL
**195**	12.88	1137.6071	–H, +HCOO	$C_{54}H_{92}O_{22}$	0.86	338.3659	1	1091.6000, 945.5448, 783.4900, 765.4748, 621.4406, 459.3839	gypenoside V or isomer（PPD-2Glc-Xyl-GlurA）	PQL、PGL
**196**	12.91	1295.6277	–H	$C_{61}H_{100}O_{29}$	0.01	364.1287	0	1133.5045, 945.5422, 621.4376, 443.3537	$C_{30}H_{52}O_2$-3Glc-Rha-2Bute-2Ace	PNL
**197**	12.92	1087.5325	–H	$C_{53}H_{84}O_{23}$	–0.45	363.9683	1	925.4783, 731.4347, 551.3727, 455.3517	stipuleanoside R2 or isomer（OA-GlurA-2Glc-Xyl）	PNL、PQL
**198**	12.93	941.5118	–H	$C_{48}H_{78}O_{18}$	0.34	316.5504	0	925.4802, 795.4447, 551.3751, 459.3838	isomer of calenduloside-B（PPD-Glc-Rha-Xyl-Ace）	PQL
**199**	12.97	1105.5828	–H	$C_{54}H_{90}O_{23}$	2.55	341.4020	1	915.5359, 459.3832, 375.2900	5,6-didehydroginsenoside Rb1 or isomer（PPD-2Glc-Rha-Xyl-CO_2）	PGL、PNL
**200**[a]	12.98	1163.5855	–H	$C_{56}H_{92}O_{25}$	0	354.5736	1	1119.5955, 1077.5850, 1059.5750, 945.5428, 783.4904, 621.4368, 459.3839, 375.2869	m-ginsenoside Rb2	PGL、PNL、PQL
**201**	13.06	943.5286	–H	$C_{48}H_{80}O_{18}$	1.49	323.5357	1	781.4736, 441.3702	ginsenoside Rh18 or isomer（$C_{30}H_{50}O_2$-GlurA-Glc-Rha-H_2O）	PGL
**202**	13.13	1193.5963	–H	$C_{57}H_{94}O_{26}$	0.24	357.7584	1	1149.6023, 1107.5951, 1089.5881, 945.5432, 927.5298, 783.4909, 621.4324, 459.3777	isomer of m-ginsenoside Rb1（PPD-4Glc-Mal）	PNL、PQL
**203**[a]	13.21	683.4367	–H, +HCOO	$C_{36}H_{62}O_9$	–1.39	271.9206	1	683.4367, 637.4324, 529.2986, 475.3788	ginsenoside F1	PGL、PNL
**204**	13.22	737.3542	–H	$C_{41}H_{54}O_{12}$	–0.03	287.5583	0	561.1876, 475.3788	PPT-GlurA-Mal	PGL
**205**	13.30	1105.5786	–H	$C_{54}H_{90}O_{23}$	–1.26	339.1267	1	967.4445, 897.5241, 837.4105, 605.1036, 537.3440, 459.3837	5,6-didehydroginsenoside Rb1 or isomer（PPD-GlurA-2Glc-Rha）	PNL、PQL
**206**	13.32	1163.5853	–H	$C_{56}H_{92}O_{25}$	–0.2	344.7831	1	1119.5942, 1077.5842, 1059.5744, 945.5404, 783.4889, 621.4363, 459.3831, 375.2867	isomer of m-ginsenoside Rc（PPD-3Glc-Xyl-Mal）	PGL、PNL、PQL
**207**	13.36	1059.5373	–H	$C_{52}H_{84}O_{22}$	–0.73	252.3240	0	955.4916, 793.4420, 455.3532	OA-GlurA-2Glc-Ace-CO_2-H_2O	PQL
**208**	13.38	1091.5644	–H	$C_{53}H_{88}O_{23}$	0.08	343.4711	1	1091.5617, 933.4142, 839.4105, 573.3251, 459.9779	Notoginsenoside Ng2 or isomer（$C_{30}H_{52}O_3$-3Glc-Rha）	PQL
**209**	13.40	955.4913	–H	$C_{48}H_{76}O_{19}$	0.52	334.1731	1	835.4539, 793.4381, 731.4378, 569.3855, 455.3521	chikusetsusaponin V or isomer（OA-2Glc-GlurA）	PQL
**210**	13.40	725.4487	–H, +HCOO	$C_{38}H_{64}O_{10}$	0.83	285.8828	0	573.1645, 441.2354	$C_{30}H_{50}O_2$-Xyl-2CO_2	PQL
**211**	13.42	1093.5808	–H, +HCOO	$C_{52}H_{88}O_{21}$	0.75	334.9917	1	1047.5763, 915.5352, 783.4894, 753.4815, 621.4387, 459.3865, 375.3030	gypenoside XV or isomer（PPD-2Glc-2Xyl）	PGL、PNL
**212**	13.48	1295.6301	–H	$C_{61}H_{100}O_{29}$	1.81	374.4634	0	1251.6348, 1209.6262, 1191.6170, 1077.5867, 1059.5767, 915.5288, 783.4895, 621.4349, 459.3835	PPD-2Glc-2Rha-Xyl-2CO_2	PNL

**213**	13.54	1165.6006	–H, +HCOO	$C_{55}H_{92}O_{23}$	–0.44	366.0178	1	1119.6007, 1077.5852, 1059.5759, 945.5440, 915.5366, 783.4919, 765.4775, 621.4390, 459.3945	pseudoginsenoside F8 or isomer（PPD-3Glc-Xyl-Ace）	PNL、PQL
**214**	13.75	1091.5647	–H	$C_{53}H_{88}O_{23}$	0.41	341.1041	1	969.4484, 837.4073, 717.3620, 537.3440, 441.3749	Notoginsenoside Ng2 or isomer（$C_{30}H_{50}O_2$-3Glc-Rha-H_2O）	PQL
**215**	13.76	1163.5856	–H	$C_{56}H_{92}O_{25}$	0.06	338.4176	1	1119.5957, 1077.5845, 1059.5724, 945.5421, 783.4905, 621.4367, 459.3835, 375.2900	isomer of m-ginsenoside Rc（PPD-3Glc-Xyl-Mal）	PGL、PNL、PQL
**216**	13.80	1105.5796	–H	$C_{54}H_{90}O_{23}$	–0.32	339.4126	1	783.4898, 621.4380, 459.3857, 375.2901	epoxynotoginsenoside A or isomer（PPD-GlurA-2Glc-Rha）	PNL
**217**	14.17	1107.5606	–H	$C_{53}H_{88}O_{24}$	1.14	341.6669	0	945.5423, 783.4896, 621.4366, 459.3839	PPD-4Glc	PQL
**218[a]**	14.19	945.5424	–H	$C_{48}H_{82}O_{18}$	–0.41	325.4825	1	765.4783, 605.4077, 537.3428, 457.3662, 373.3141	ginsenoside Rd	PGL、PNL、PQL
**219**	14.22	983.5212	–H	$C_{50}H_{80}O_{19}$	–0.91	321.7443	0	821.4058, 591.9057, 459.3075, 375.2899	isomer of chikusetsusaponin V ethyl ester（PPD-Glc-Rha-Xyl-2Ace）	PGL、PNL
**220[a]**	14.27	925.4771	–H	$C_{47}H_{74}O_{18}$	–3.38	313.8303	1	735.3794, 605.4076, 557.2633, 439.3570	pseudoginsenoside Rt1	PQL
**221**	14.28	1165.6013	–H, +HCOO	$C_{55}H_{92}O_{23}$	0.2	364.4360	1	1119.5960, 1077.5851, 1059.5756, 945.5430, 783.4901, 621.4378, 459.3823	pseudoginsenoside F8 or isomer（PPD-3Glc-Xyl-Ace）	PNL、PQL
**222**	14.31	1093.5790	–H	$C_{53}H_{90}O_{23}$	–0.94	348.3157	1	885.2979, 753.4891, 459.4338	floranotoginsenoside A or isomer（$C_{30}H_{52}O_3$-3Glc-Xyl）	PGL、PNL
**223**	14.32	1163.5861	–H	$C_{56}H_{92}O_{25}$	0.47	347.1183	1	1119.5930, 1077.5844, 1059.5760, 945.5396, 783.4908, 621.4370, 459.3839, 375.2839	isomer of m-ginsenoside Rc（PPD-3Glc-Xyl-Mal）	PNL
**224**	14.33	855.4756	–H	$C_{44}H_{72}O_{16}$	0.97	288.8792	1	729.7736, 571.3050, 373.2745	notoginsenosides NL-D or isomer（5,6-didehydro-PPD-Glc-Xyl-Mal-2Ace-H_2O）	PNL
**225**	14.35	943.5273	–H	$C_{48}H_{80}O_{18}$	0.1	321.4684	1	855.4280, 837.4045, 705.3714, 459.4338	ginsenoside Rh18 or isomer（$C_{30}H_{52}O_3$-Glc-Xyl-2CO_2-2Ace-H_2O）	PGL
**226**	14.42	991.5484	–H, +HCOO	$C_{48}H_{82}O_{18}$	0.09	328.3004	1	945.5405, 783.4899, 621.4367, 459.3832, 375.2912	chikusetsusaponin FK7 or isomer（PPD-3Glc）	PGL
**227**	14.56	1165.6003	–H, +HCOO	$C_{55}H_{92}O_{23}$	–0.67	353.0541	1	1119.5951, 1077.5840, 945.5423, 783.4901, 765.4793, 621.4356, 459.3818, 375.2875	pseudoginsenoside F8 or isomer（PPD-3Glc-Xyl-Ace）	PGL、PNL、PQL
**228[a]**	14.63	973.4108	–H	$C_{49}H_{66}O_{20}$	3.51	322.1324	0	811.4853, 621.4356, 459.9472	$C_{30}H_{52}O_3$-2Glc-Dimal-H_2O	PNL
**229**	14.63	857.4907	–H, +HCOO	$C_{43}H_{72}O_{14}$	0.31	308.8542	1	811.4840, 631.3898, 517.3916, 433.2976, 391.2958	20(*S*)-sanchirhinoside A2 or isomer（$C_{24}H_{40}O_4$-Glc-Rha-Bute-CO_2）	PNL
**230**	14.64	1031.5439	–H	$C_{51}H_{84}O_{21}$	0.72	336.7822	1	943.5255, 925.5184, 781.4746, 619.4170, 457.3686	m-ginsenoside Rd1 or isomer（$C_{30}H_{50}O_3$-2Glc-Rha-Mal-H_2O）	PGL
**231**	14.64	855.4754	–H	$C_{44}H_{72}O_{16}$	0.83	289.8556	1	811.4853, 621.4357, 459.2648	notoginsenosides NL-D or isomer（PPD-2Rha-Mal-H_2O）	PNL
**232**	14.64	955.3970	–H	$C_{49}H_{64}O_{19}$	0.06	301.2727	1	631.3357, 455.3632	chikusetsusaponin V or isomer（OA-GlurA-2Glc）	PNL
**233**	14.69	1093.5794	–H, +HCOO	$C_{52}H_{88}O_{21}$	–0.53	343.3002	1	1047.5712, 915.5320, 753.4792, 621.4362, 459.3808, 375.2943	gypenoside XV or isomer（PPD-2Glc-2Xyl）	PGL、PNL
**234**	14.73	723.4334	–H, +HCOO	$C_{38}H_{62}O_{10}$	1.39	280.3893	0	527.2868, 459.3855	PPD-Xyl-Bute-H_2O	PGL
**235**	14.77	1163.5862	–H	$C_{56}H_{92}O_{25}$	0.61	365.6328	1	1119.6000, 1077.5857, 1059.5762, 945.5416, 783.4902, 765.4790, 621.4381, 459.3848, 375.2966	isomer of m-ginsenoside Rc（PPD-3Glc-Xyl-Mal）	PGL、PNL

**236**	14.92	1249.5864	–H	$C_{59}H_{94}O_{28}$	0.41	354.8976	0	917.3033, 717.3704, 603.4327, 459.3847, 375.2899	PPD-3Glc-Dimal-Xyl	PNL、PQL
**237**	15.02	991.5480	–H, +HCOO	$C_{48}H_{82}O_{18}$	–0.33	330.2518	1	945.5413, 783.4893, 621.4351, 459.3855	ginsenoside Rg18 or isomer（PPD-3Glc）	PGL
**238**	15.20	1165.5992	–H, +HCOO	$C_{55}H_{92}O_{23}$	–1.69	352.7174	1	1119.5954, 1077.5850, 1059.5738, 945.5424, 783.4886, 621.4359, 459.3836, 375.2887	pseudoginsenoside F8 or isomer（PPD-3Glc-Xyl-Ace）	PQL、PGL
**239**	15.22	1105.5814	–H	$C_{54}H_{90}O_{23}$	1.22	339.0706	1	1059.5734, 927.5333, 765.4804, 603.4309, 441.3734, 373.3116	epoxynotoginsenoside A or isomer（$C_{30}H_{50}O_2$-GlurA-2Glc-Rha-H_2O）	PNL
**240**	15.24	925.4798	–H	$C_{47}H_{74}O_{18}$	–0.39	322.0344	1	763.4299, 455.3537	isomer of pseudoginsenoside Rt1（OA-2Glc-Rha）	PQL
**241**	15.25	1163.5855	–H	$C_{56}H_{92}O_{25}$	0.04	360.4723	1	1119.5934, 1077.5842, 1059.5747, 945.5421, 783.4906, 621.4362, 459.3846, 375.2916	isomer of m-ginsenoside Rc（PPD-3Glc-Xyl-Mal）	PGL、PNL
**242**	15.29	991.5513	–H, +HCOO	$C_{48}H_{82}O_{18}$	3.16	322.6056	1	945.5396, 783.4905, 621.4367, 459.3890, 375.2864	chikusetsusaponin FK7 or isomer（PPD-3Glc）	PGL
**243**	15.34	961.5371	–H	$C_{48}H_{82}O_{19}$	–0.62	316.4598	1	785.4966, 571.3987, 501.3960, 443.3528, 375.2898	quinquenoside L2 or isomer（PPD-Glc-Rha-Xyl-CO_2-H_2O）	PQL
**244**	15.35	987.5537	–H	$C_{50}H_{84}O_{19}$	0.3	331.3449	1	747.3850, 675.3564, 537.3431, 459.3844	6″-*O*-acetylginsenoside Rg3 or isomer（PPD-3Glc-Ace）	PQL
**245**	15.36	1035.5642	–H, +HCOO	$C_{57}H_{82}O_{14}$	–4.53	340.1807	0	827.2967, 459.3844	PPD-3Glc-CO_2	PQL
**246**[a]	15.36	1031.5437	–H	$C_{51}H_{84}O_{21}$	0.49	335.7891	1	997.4630, 833.4122, 707.3733, 537.3431, 459.3844	m-ginsenoside Rd	PGL、PNL、PQL
**247**	15.39	815.4804	–H	$C_{42}H_{72}O_{15}$	0.68	295.9323	1	783.4903, 621.4367, 459.3844	floralginsenoside F or isomer（PPD-Glc-Xyl-CO_2-H_2O）	PQL
**248**	15.48	691.3543	–H	$C_{33}H_{56}O_{15}$	–0.36	267.8235	0	457.3672	$C_{30}H_{50}O_3$-Xyl-2Ace-H_2O	PGL
**249**	15.49	1205.5940	–H	$C_{58}H_{94}O_{26}$	–1.65	363.4017	0	1119.5960, 1077.5811, 1059.5736, 945.5462, 765.4857, 621.4346, 459.3861, 375.2870	PPD-3Glc-Xyl-Mal-Ace	PQL
**250**	15.64	665.4272	–H	$C_{36}H_{62}O_{10}$	0.26	262.8122	1	475.3791, 391.2849	3-formyloxy-20-*O*-*β*-D-glucopyranosyl-20(*S*)-protopanaxtriol or isomer（PPT-Rha-CO_2）	PGL
**251**	15.65	679.4433	–H	$C_{38}H_{64}O_{10}$	1.1	274.6643	1	637.4320, 475.3791, 391.2849	20(*S*)-ginsenoside-Rh1-6'-acetate or isomer（PPT-Glc-Ace）	PGL
**252**	15.70	1031.5429	–H	$C_{51}H_{84}O_{21}$	–0.33	330.3502	1	945.5427, 783.4959, 621.4353, 459.3784	m-ginsenoside Rd1 or isomer（PPD-3Glc-Mal）	PGL
**253**	15.72	1165.6004	–H, +HCOO	$C_{55}H_{92}O_{23}$	–0.61	366.6294	1	1119.5975, 1077.5842, 1059.5744, 945.5428, 783.4905, 765.4800, 621.4275, 459.3827, 375.2988	pseudoginsenoside F8 or isomer（PPD-3Glc-Xyl-Ace）	PGL、PNL、PQL
**254**[a]	15.77	1163.5844	–H	$C_{56}H_{92}O_{25}$	–0.93	354.9716	1	733.3892, 649.3070, 459.3835	m-ginsenoside Rc	PNL、PQL
**255**	15.83	961.5374	–H, +HCOO	$C_{47}H_{80}O_{17}$	–0.44	313.1629	1	915.5354, 621.4343, 459.3847	quinquenoside L10 or isomer（PPD-2Glc-Xyl）	PQL
**256**[a]	15.92	945.5420	–H	$C_{48}H_{82}O_{18}$	–0.89	318.6634	1	793.4351	Gypenoside XVII	PGL、PNL、PQL
**257**[a]	15.95	793.4385	–H	$C_{42}H_{66}O_{14}$	0.64	284.9517	1	455.3526, 423.3259	chikusetsusaponin Iva	PQL、PGL
**258**	16.07	1165.6028	–H, +HCOO	$C_{55}H_{92}O_{23}$	1.47	357.7315	1	1119.5983, 1077.5847, 1059.5765, 945.5432, 783.4893, 621.4381, 459.3846	pseudoginsenoside F8 or isomer（PPD-3Glc-Xyl-Ace）	PNL、PQL
**259**	16.14	1033.5587	–H, +HCOO	$C_{50}H_{84}O_{19}$	–0.18	337.0223	1	987.5543, 945.5434, 783.4895, 621.4365, 459.3842, 375.2884	6″-*O*-acetylginsenoside Rg3 or isomer（PPD-3Glc-Ace）	PGL、PNL、PQL
**260**	16.16	1075.5690	–H	$C_{53}H_{88}O_{22}$	–0.38	332.3759	1	621.4337, 441.3712	quinquefoloside-Lb or isomer（$C_{30}H_{50}O_2$-2Glc-2Rha-H_2O）	PQL
**261**	16.17	959.5222	–H	$C_{48}H_{80}O_{19}$	0.07	313.0161	1	779.4584, 621.4380, 459.3817	vinaginsenoside R20 or isomer（PPD-GlurA-2Glc）	PQL

**262**	16.21	1031.5430	–H	$C_{51}H_{84}O_{21}$	–0.19	319.3662	1	621.4370, 459.3115, 375.2902	m-ginsenoside Rd1 or isomer（PPD-3Glc-Mal）	PGL、PNL、PQL
**263**	16.36	679.4426	–H	$C_{38}H_{64}O_{10}$	0.07	281.5844	1	517.1345, 391.2864	20(*S*)-ginsenoside-Rh1-6'-acetate or isomer（PPT-Glc-3Ace）	PGL
**264**	16.57	987.5536	–H	$C_{50}H_{84}O_{19}$	0.23	339.9146	1	783.4935, 459.3834, 375.2896	6″-*O*-acetylginsenoside Rg3 or isomer（PPD-3Glc-Ace）	PQL
**265**	16.68	1075.5688	–H	$C_{53}H_{88}O_{22}$	–0.57	330.0632	1	1075.5680, 763.4547, 441.3621	quinquefoloside-Lb or isomer（$C_{30}H_{50}O_2$-2Glc-2Rha-H_2O）	PQL
**266**	16.71	1093.5803	–H, +HCOO	$C_{52}H_{88}O_{21}$	0.27	346.2276	1	1047.5745, 915.5314, 783.4888, 621.4353, 459.3842, 375.2888	gypenoside XV or isomer（PPD-2Glc-2Xyl）	PNL
**267**	16.92	1117.5435	–H	$C_{54}H_{86}O_{24}$	–0.08	342.6260	1	987.5579, 945.5402, 927.5345, 879.5172, 783.4887, 621.4367, 459.3850	malonylfloralginsenoside Rd6 or isomer（PPD-3Glc-Dimal）	PQL
**268**	17.14	1165.5998	–H, +HCOO	$C_{55}H_{92}O_{23}$	–1.2	353.1780	1	1119.5889, 1077.5830, 1059.5736, 945.5394, 783.4910, 621.4380, 459.3834	pseudoginsenoside F8 or isomer（PPD-3Glc-Xyl-Ace）	PNL
**269**	17.16	1071.5736	–H	$C_{54}H_{88}O_{21}$	–0.87	339.9531	1	945.5414, 783.4946, 621.4410, 459.3836	ginsenoside Rh25 or isomer（PPD-2Glc-Xyl-Bute-2CO_2）	PGL
**270**	17.29	1033.5595	–H, +HCOO	$C_{50}H_{84}O_{19}$	0.56	337.8342	1	987.5564, 945.5432, 927.5307, 783.4904, 765.4816, 621.4374, 459.3836, 375.2856	6″-*O*-acetylginsenoside Rg3 or isomer（PPD-3Glc-Ace）	PGL、PNL、PQL
**271**	17.29	725.4490	–H, +HCOO	$C_{38}H_{64}O_{10}$	1.29	282.5953	0	503.2506, 459.3867	PPD-Xyl-CO_2	PGL
**272**	17.31	1117.5431	–H	$C_{54}H_{86}O_{24}$	–0.44	336.5106	1	987.5542, 945.5429, 921.5207, 825.5012, 765.4798, 621.4360, 459.3845, 375.2896	malonylfloralginsenoside Rd6 or isomer（PPD-3Glc-Dimal）	PGL、PNL、PQL
**273**	17.32	1075.5688	–H, +HCOO	$C_{52}H_{86}O_{20}$	–0.54	354.1174	0	909.5181, 825.5043, 705.3525, 459.3849	PPD-3Glc-2Ace	PGL
**274**	17.35	653.4268	–H	$C_{36}H_{62}O_{10}$	–0.33	264.4534	1	621.4383, 459.3848	ginsenoside M7cd or isomer（PPD-GlurA-H_2O）	PGL
**275[a]**	17.37	961.5378	–H, +HCOO	$C_{47}H_{80}O_{18}$	–0.02	317.2583	1	783.4901, 621.4366, 459.3833, 375.2892	notoginsenoside Fe	PGL、PNL、PQL
**276**	17.39	945.5411	–H	$C_{48}H_{82}O_{18}$	–1.78	324.2638	1	783.6360, 621.4380, 459.3849, 375.2901	chikusetsusaponin FK7 or isomer（PPD-3Glc）	PGL
**277**	17.46	1031.5429	–H	$C_{51}H_{84}O_{21}$	–0.29	331.5339	1	945.5413, 927.5311, 783.4884, 621.4356, 603.4295, 459.3826, 375.2905	m-ginsenoside Rd1 or isomer（PPD-3Glc-Mal）	PGL、PNL、PQL
**278**	17.47	1091.5604	–H	$C_{53}H_{88}O_{23}$	–3.56	339.7215	1	673.3501, 475.3078, 375.2896	Notoginsenoside Ng2 or isomer（$C_{24}H_{40}O_3$-3Glc-Rha-2Ace）	PQL
**279**	17.47	815.4791	–H	$C_{42}H_{72}O_{15}$	–0.85	290.2999	1	571.3991, 375.1972	floralquinquenoside B or isomer（$C_{24}H_{40}O_3$-GlurA-Glc-2Ace-H_2O）	PQL
**280**	17.49	829.4950	–H	$C_{43}H_{74}O_{15}$	–0.65	301.9290	1	537.3450, 439.3595, 375.1972	notoginsenoside ST-2 or isomer（$C_{24}H_{40}O_3$-Glc-2Rha）	PQL
**281**	17.53	975.5179	–H, +HCOO	$C_{47}H_{78}O_{18}$	0.95	318.1824	1	767.3480, 621.4366, 441.3712	notoginsenosides NL-C1 or isomer（$C_{30}H_{50}O_2$-2Glc-Rha-H_2O）	PQL
**282**	17.56	1181.6998	–H	$C_{66}H_{102}O_{18}$	0.39	264.0835	0	695.3613, 475.3786	PPT-3Glc-Xyl-2CO_2	PNL
**283**	17.56	679.4426	–H	$C_{38}H_{64}O_{10}$	–0.11	279.7462	0	591.3326, 459.3704, 375.2899	isomer of 6'-acetyl ginsenoside-F1（PPD-Xyl-2CO_2-2Ace）	PNL
**284**	17.56	683.4376	–H, +HCOO	$C_{36}H_{62}O_9$	0	268.4041	1	475.3786, 391.2851	ginsenoside Rh13 or isomer（PPT-Glc-2Ace）	PNL
**285**	17.56	665.4274	–H	$C_{37}H_{62}O_{10}$	0.63	262.5117	1	475.3786	3-formyloxy-20-*O*-*β*-D-glucopyranosyl-20(*S*)-protopanaxtriol or isomer（PPT-Rha-CO_2）	PNL
**286**	17.56	769.4384	–H, +HCOO	$C_{39}H_{64}O_{12}$	0.49	280.0159	0	591.3326, 459.3704, 375.2899	PPD-2Xyl	PNL
**287**	17.60	693.3700	–H	$C_{33}H_{58}O_{15}$	–0.49	267.9330	0	697.3796, 475.3786, 391.2851	PPT-Xyl-Bute-H_2O	PNL

**288**	17.60	953.5124	–H	$C_{49}H_{78}O_{18}$	0.99	313.7691	0	765.4750, 605.4070, 459.2096, 441.3712, 375.2896	isomer of taibaienoside II（$C_{30}H_{50}O_2$-GlueA-Glc-Xyl-Ace）	PQL
**289**[a]	17.61	961.5385	–H, +HCOO	$C_{47}H_{80}O_{17}$	0.82	317.5831	1	915.5319, 783.4903, 621.4372, 459.3846	notoginsenoside Fd/ginsenoside Rd2	PGL、PNL、PQL
**290**	17.63	941.5133	–H	$C_{48}H_{78}O_{18}$	1.93	314.1139	0	605.4070, 459.3896, 375.2902	isomer of calenduloside-B（PPD-GlurA-2Xyl-Ace）	PQL
**291**	17.64	809.4690	–H	$C_{43}H_{70}O_{14}$	–0.39	296.6541	0	637.4304, 391.2856	PPT-Glc-2Mal-2Ace	PGL
**292**	17.64	1121.5741	–H, +HCOO	$C_{53}H_{88}O_{22}$	–0.74	340.4608	1	915.5327, 783.4897, 621.4368, 459.3842	quinquefoloside-Lb or isomer（PPD-2Glc-2Rha）	PGL、PNL、PQL
**293**	17.65	1089.5496	–H	$C_{53}H_{86}O_{23}$	0.85	338.3637	0	1043.5599, 915.5327, 783.4897, 621.4368, 459.3842	PPD-2Glc-2Xyl-Ace	PQL
**294**	17.66	1117.5436	–H	$C_{54}H_{86}O_{24}$	–0.02	346.3432	1	945.5402, 783.4764, 621.4420, 459.3792, 375.2924	malonylfloralginsenoside Rd6 or isomer（PPD-3Glc-Dimal）	PGL、PNL、PQL
**295**	17.66	943.5266	–H	$C_{48}H_{80}O_{18}$	–0.65	327.5276	0	915.5327, 767.8154, 621.4368, 605.4084, 459.3842	isomer of quinquenoside L1（PPD-GlurA-Glc-Rha）	PQL、PGL
**296**	17.66	1073.5543	–H	$C_{53}H_{86}O_{22}$	0.47	336.4461	0	987.5547, 783.4897, 621.4368, 459.3842	PPD-3Glc-Mal-Ace	PQL
**297**	17.68	1001.5311	–H	$C_{50}H_{82}O_{20}$	–1.49	330.9395	0	915.5327, 375.2900	$C_{24}H_{40}O_3$-2Glc-Xyl-Mal-2Ace	PQL、PGL
**298**	17.69	1003.5460	–H, +HCOO	$C_{49}H_{82}O_{18}$	–2.43	331.9447	0	783.4920, 459.3842	PPD-2Glc-Xyl-Ace	PGL、PNL、PQL
**299**	17.69	829.4938	–H, +HCOO	$C_{42}H_{72}O_{13}$	–2.1	301.1828	1	783.4901, 375.2902	ginsenoside F2 or isomer（$C_{24}H_{40}O_3$-2Glc-2Ace）	PQL
**300**	17.70	1107.5961	–H, +HCOO	$C_{53}H_{90}O_{21}$	0.06	347.8494	0	917.5388, 765.4771, 665.3141, 737.5347, 459.3842	PPD-2Glc-Rha-Xyl	PNL
**301**	17.72	945.5402	–H	$C_{48}H_{82}O_{18}$	–2.77	321.3455	1	853.4433, 771.4500, 615.3455, 375.29	isomer of ginsenoside Rd（$C_{24}H_{40}O_3$-3Glc）	PGL
**302**	17.73	813.4644	–H	$C_{42}H_{70}O_{15}$	0.29	309.7221	1	771.4500, 615.3455, 475.3791	isomer of pseudoginsenoside RT8（PPT-GlurA-Glc）	PGL
**303**	17.77	827.4780	–H	$C_{43}H_{72}O_{15}$	–2.23	293.8471	0	783.4883, 739.4241, 721.4133, 457.3691	isomer of vinaginsenoside R2（$C_{30}H_{50}O_3$-2Xyl-H_2O-2CO_2）	PGL
**304**	17.77	1033.5580	–H, +HCOO	$C_{50}H_{84}O_{19}$	–0.86	336.7252	1	987.5506, 945.5476, 927.5287, 783.4839, 765.4836, 621.4378, 459.3797, 375.2931	6″-O-acetylginsenoside Rg3 or isomer（PPD-3Glc-Ace）	PGL、PNL、PQL
**305**[a]	17.83	811.4844	–H, +HCOO	$C_{42}H_{70}O_{12}$	–0.69	297.0436	1	739.4230, 631.3267, 531.3124, 391.9970	ginsenoside Rg6	PGL、PNL、PQL
**306**	17.84	975.5519	–H, +HCOO	$C_{48}H_{82}O_{17}$	–1.59	325.6355	1	929.5470, 783.4909, 621.4379, 459.3862, 375.2914	vinaginsenoside R3 or isomer（PPD-2Glc-Rha）	PGL、PNL
**307**	17.85	959.5234	–H	$C_{48}H_{80}O_{19}$	1.36	316.8746	1	783.4895, 375.2897	vinaginsenoside R20 or isomer（$C_{24}H_{40}O_3$-GlurA-2Glc-2Ace）	PGL
**308**	17.87	941.5114	–H	$C_{48}H_{78}O_{18}$	–0.13	321.2803	0	677.8905, 459.384	isomer of calenduloside-B（PPD-GlurA-2Xyl-Ace）	PQL
**309**	17.87	725.4474	–H	$C_{39}H_{66}O_{12}$	–0.93	280.7044	0	638.3926, 475.3781, 391.2849	PPT-Glc-2CO_2	PGL
**310**	17.87	683.4382	–H	$C_{37}H_{64}O_{11}$	0.91	272.7090	0	621.4378, 459.3838, 375.2912	isomer of notoginsenoside SP20（PPD-Glc-CO_2-H_2O）	PQL
**311**	17.90	1087.5340	–H	$C_{53}H_{84}O_{23}$	0.92	331.2390	0	957.5370, 765.4775, 621.4358, 459.3870, 375.2921	isomer of stipuleanoside R2（PPD-2Glc-Xyl-Dimal）	PNL
**312**	17.91	767.4587	–H, +HCOO	$C_{40}H_{66}O_{11}$	0.01	292.1343	1	679.4468, 459.3830	dammar-12,24-dien-3α,6β, 15α-triol-3α-arabinopyranosyl-6β-L-arabinopyranoside（PPD-Glc-Rha）	PNL
**313**	17.91	871.4694	–H	$C_{44}H_{72}O_{17}$	–0.33	286.8084	1	671.2839, 459.3830	notoginsenosides NL-A4 or isomer（PPD-Glc-Rha-Mal-3H_2O）	PNL

**314**[a]	17.91	829.4948	–H	$C_{43}H_{74}O_{15}$	–0.81	303.1545	1	701.4215, 621.4369, 459.3813, 375.3023	ginsenoside F2	PGL、PNL、PQL
**315**[a]	17.92	811.4820	–H, +HCOO	$C_{42}H_{70}O_{12}$	–3.78	301.9209	1	783.4894, 673.3487, 537.3417, 459.3840	(20*E*)-ginsenoside F4	PQL、PGL
**316**	18.04	1077.5500	–H	$C_{52}H_{86}O_{23}$	1.21	334.2100	0	1077.5547, 947.5231, 843.2321, 689.3579, 441.3721	$C_{30}H_{50}O_2$-3Glc-CO_2	PQL
**317**[a]	18.04	665.4270	–H, +HCOO	$C_{36}H_{60}O_8$	0.04	275.9321	1	617.2843, 561.2546, 407.2454	ginsenoside Rh4	PGL、PNL、PQL
**318**[a]	18.06	961.5372	–H, +HCOO	$C_{47}H_{80}O_{17}$	–0.67	325.2119	1	915.5348, 783.4882, 621.4334, 459.3860, 375.2844	notoginsenoside Ft1	PNL
**319**[a]	18.25	829.4942	–H, +HCOO	$C_{42}H_{72}O_{13}$	–1.68	301.7299	1	783.4924, 621.4390, 537.3399, 459.3833, 375.2882	20(*S*)-ginsenoside Rg3/20(*R*)-ginsenoside Rg3	PGL、PNL、PQL
**320**	18.33	751.4656	–H	$C_{41}H_{68}O_{12}$	2.37	292.6197	1	587.3456, 443.3528, 355.2621	notoginsenoside-LY or isomer（$C_{30}H_{52}O_2$-Glc-Rha）	PGL
**321**	18.35	871.5067	–H, +HCOO	$C_{44}H_{74}O_{14}$	0.77	309.2536	1	825.4988, 783.4892, 621.4362, 459.3836, 375.2952	20(*S*)-6'-*O*-acetyl-ginsenoside Rg2 or isomer（PPD-2Glc-2CO_2）	PQL、PGL
**322**	18.38	869.4903	–H	$C_{45}H_{74}O_{16}$	–0.15	290.7563	0	807.3026, 675.0103, 589.3029, 443.3576	$C_{30}H_{52}O_2$-Rha-Xyl-Mal-CO_2-H_2O	PNL
**323**	18.42	925.4796	–H	$C_{47}H_{74}O_{18}$	–0.64	334.8115	1	793.4374, 745.4217, 455.3518	isomer of pseudoginsenoside Rt1（OA-2Glc-Rha）	PQL
**324**	18.42	799.4855	–H	$C_{42}H_{72}O_{14}$	0.78	302.0369	0	753.4813, 459.3847, 375.2904	isomer of ginsenoside Rh20（PPD-Glc-Xyl）	PNL、PQL
**325**	18.49	793.4381	–H	$C_{42}H_{66}O_{14}$	0.16	305.9894	1	613.3765, 569.3851, 455.3534	isomer of chikusetsusaponin IVa（OA-Glc-GlurA）	PQL、PGL
**326**	18.51	955.4909	–H	$C_{48}H_{76}O_{19}$	0.1	314.5520	0	545.2836, 459.3830	isomer of chikusetsusaponin V（PPD-2Glc-Dimal）	PNL
**327**	18.59	841.4944	–H	$C_{44}H_{74}O_{15}$	–1.26	312.5749	1	501.3934, 459.3830	notoginsenoside Rt or isomer（PPD-GlurA-Rha-Ace-H_2O）	PNL
**328**[a]	18.59	943.5259	–H	$C_{48}H_{80}O_{18}$	–1.32	343.0411	1	765.4785, 717.3825, 577.2686, 533.1749	5,6-didehydroginsenoside Rd	PNL
**329**	18.69	925.4799	–H	$C_{47}H_{74}O_{18}$	–0.38	319.9587	1	709.3633, 475.3395	elatoside A or isomer（OA-2Glc-Rha)	PNL
**330**	18.76	945.5069	–H	$C_{47}H_{78}O_{19}$	0.49	314.6011	0	917.5309, 685.0311, 455.0812	$C_{30}H_{48}O_3$-Glc-2Rha-2H_2O	PQL
**331**	18.77	1033.6502	–H	$C_{50}H_{98}O_{21}$	–2.5	338.0667	0	947.6453, 945.5586, 705.4099, 475.3732	PPT-3Glc-2CO_2	PGL
**332**[a]	18.84	811.4853	–H, +HCOO	$C_{42}H_{70}O_{12}$	0.51	317.9241	1	727.3281, 513.3023, 337.2050, 313.2515	ginsenoside Rg5/ginsenoside Rk1	PGL、PNL、PQL
**333**	18.84	793.4393	–H	$C_{42}H_{66}O_{14}$	1.67	310.5840	1	569.3837, 551.3738, 455.3530	isomer of chikusetsusaponin IVa（OA-Glc-GlurA）	PQL
**334**[a]	18.84	667.4432	–H	$C_{37}H_{64}O_{10}$	0.85	271.9445	1	647.0196, 459.0484	20(*R*)-ginsenoside Rh2/compound K	PGL、PNL、PQL
**335**	18.94	813.4671	–H	$C_{42}H_{70}O_{15}$	3.5	300.1601	1	545.4661, 459.1716	pseudoginsenoside RT8 or isomer（$C_{22}H_{36}O_{10}$-Glc-Rha）	PQL
**336**	18.99	603.3384	–H	$C_{30}H_{52}O_{12}$	–0.36	251.5626	0	441.4910	$C_{30}H_{50}O_2$-Glc	PNL
**337**	19.06	763.4271	–H	$C_{41}H_{64}O_{13}$	–0.33	304.7133	1	551.3680, 455.3559	pseudoginsenoside RP1 or isomer（OA-Glc-Rha）	PQL
**338**	19.74	1015.5854	–H, +HCOO	$C_{51}H_{86}O_{17}$	0.67	331.1417	0	865.1393, 653.3044, 441.2674	$C_{30}H_{50}O_2$-2Glc-Rha-Bute-2H_2O	PNL
**339**	19.93	1075.6947	–H	$C_{60}H_{100}O_{16}$	0.85	347.7870	0	1058.6321, 991.6129, 751.4808, 637.4514, 475.2714, 391.2850	PPT-2Glc-Rha-Mal-CO_2	PGL
**340**	20.00	997.5755	–H	$C_{52}H_{86}O_{18}$	1.36	328.0450	0	645.2955, 457.2024	$C_{30}H_{50}O_3$-2Glc-Xyl-2Ace	PGL
**341**	20.24	999.5906	–H, +HCOO	$C_{51}H_{86}O_{16}$	0.82	325.6197	0	941.9059, 691.3687, 391.5858	$C_{24}H_{40}O_4$-2Glc-2Bute-2Ace-H_2O	PNL
**342**	20.71	591.2618	–H	$C_{34}H_{40}O_9$	3.24	261.5674	0	515.2440, 459.2220	$C_{22}H_{36}O_{10}$-Xyl	PGL

[a]: The components identified by comparison with the reference standards.

0: The components identified manually based on the MS information as no hits were found in the in-house ginsenoside library (not reported).

1: The components identified manually based on the MS information successfully matching with the in-house ginsenoside library.

PGL: the leaf of *Panax ginseng*, PQL: the leaf of *Panax quinquefolius*, PNL: the leaf of *Panax notoginseng*.

Mal: The structure identified with a single malonyl substituent.

Dimalonyl: The structure identified with two malonyl substituents.

Ace：The structure identified with an acetyl substituent.

2Ace: The structure identified with two acetyl substituents.

Butenoyl: The structure identified with a butenoyl substituent.

Table S5 Detailed information of 42 potential ginsenoside markers diagnostic for differentiating among PGL, PQL, and PNL.

**No.**	**VIP**	***m/z***	**t_R (min)**	**Assignment**	**MS^2 fragments**	**Identification**	**PGL**	**PQL**	**PNL**
**M1[a]**	23.91	845.4904	9.79	[M–H+HCOOH]$^-$	707.3480, 635.4158, 491.3736	24(*R*)–pseudoginsenoside F11	L	H	L
**M2[a]**	22.21	991.5484	6.33	[M–H+HCOOH]$^-$	945.5427, 783.4921, 637.4324, 475.3789, 391.2829	ginsenoside Re	H	M	L
**M3[a]**	19.24	1123.5900	12.49	[M–H+HCOOH]$^-$	1077.5842, 945.5421, 783.4897, 621.4370, 459.3842, 375.2899	ginsenoside Rb3	L	H	M
**M4[a]**	18.51	683.4376	13.19	[M–H+HCOOH]$^-$	683.4367, 637.4324, 529.2986, 475.3788	ginsenoside F1	H	L	L
**M5[a]**	17.49	815.4797	11.32	[M–H+HCOOH]$^-$	815.4795, 769.4742, 529.2983, 475.3785, 391.2851	ginsenoside F3	H	L	L
**M6**	16.99	1163.5850	13.28	[M–H]$^-$	1119.5942, 1077.5842, 1059.5744, 945.5404, 783.4889, 621.4363, 459.3831, 375.2867	isomer of m–ginsenoside Rc	L	H	M
**M7**	16.09	725.4481	15.65	[M–H+HCOOH]$^-$	637.4320, 475.3791, 391.2849	20(*S*)–ginsenoside–Rh1–6'–acetate or isomer	H	L	L
**M8[a]**	15.20	815.4798	11.11	[M–H+HCOOH]$^-$	815.4803, 783.4903, 621.4373, 475.3794, 391.2856	ginsenoside F5	H	L	L
**M9[a]**	14.07	1255.6320	11.61	[M–H+HCOOH]$^-$	1001.4668, 605.4039, 459.3834	ginsenoside Ra1	L	L	H
**M10**	13.95	1033.5550	15.34	[M–H+HCOOH]$^-$	747.3850, 675.3564, 537.3431, 459.3844	6″–*O*–acetylginsenoside Rg3 or isomer	M	H	L
**M11[a]**	12.51	1007.5430	8.83	[M–H+HCOOH]$^-$	723.3535, 621.4079, 475.3685, 391.2845	vinaginsenoside R4	H	M	L
**M12[a]**	12.10	1285.6420	10.64	[M–H+HCOOH]$^-$	1077.5813, 897.5236, 783.4910, 621.4365, 459.3843, 375.2904	notoginsenoside Fa	L	L	H
**M13**	11.84	1033.5570	7.91	[M–H+HCOOH]$^-$	945.5429, 783.4896, 637.4326, 475.3794, 391.2854	pseudoginsenoside RC1 or isomer	H	L	L
**M14[a]**	11.57	961.5376	17.59	[M–H+HCOOH]$^-$	915.5319, 783.4903, 621.4372, 459.3846	notoginsenoside Fd/ginsenoside Rd2	L	M	H
**M15**	9.57	1033.5570	17.35	[M–H+HCOOH]$^-$	987.5564, 945.5432, 927.5307, 783.4904, 765.4816, 621.4374, 459.3836, 375.2856	6″–*O*–acetylginsenoside Rg3 or isomer	M	H	L
**M16[a]**	9.52	829.4953	11.85	[M–H+HCOOH]$^-$	829.44957, 783.4901, 637.4321, 475.3789, 391.2849	ginsenoside Rg2	H	M	L
**M17[a]**	9.47	1255.6320	11.08	[M–H+HCOOH]$^-$	999.4559, 879.4133, 747.3764, 537.3441, 459.3843	ginsenoside Ra2	L	L	H
**M18**	9.37	1163.5850	13.76	[M–H]$^-$	1119.5957, 1077.5845, 1059.5724, 945.5421, 783.4905, 621.4367, 459.3835, 375.2900	isomer of m–ginsenoside Rc	L	H	M
**M19**	9.24	855.3301	4.24	[M–H]$^-$	809.3235, 805.2941, 761.3037, 491.1670	OT–Rha–Xyl–Mal	M	H	L
**M20**	8.90	815.4796	11.52	[M–H+HCOOH]$^-$	475.3793, 373.2735	isomer of ginsenoside F5	H	L	L
**M21[a]**	8.54	1123.5900	11.71	[M–H+HCOOH]$^-$	1077.5851, 945.5426, 765.4794, 621.4376, 459.3844, 375.2903	ginsenoside Rc	H	L	H
**M22**	8.38	1117.5440	17.31	[M–H]$^-$	987.5542, 945.5429, 921.5207, 825.5012, 765.4798, 621.4360, 459.3845, 375.2896	malonylfloralginsenoside Rd6 or isomer	M	H	L
**M23**	7.70	863.5004	4.04	[M–H+HCOOH]$^-$	817.4963, 671.4375, 653.4265, 509.3845, 391.2847	quinquenoside L9	L	H	L
**M24[a]**	7.70	1163.5850	12.97	[M–H]$^-$	1119.5955, 1077.5850, 1059.5750, 945.5428, 783.4904, 621.4368, 459.3839, 375.2869	m–ginsenoside Rb2	M	H	L
**M25[a]**	7.61	1123.5900	12.23	[M–H+HCOOH]$^-$	1077.5832, 945.5410, 783.4892, 621.4368, 459.3837	ginsenoside Rb2	H	H	L
**M26**	7.61	1033.5570	16.18	[M–H+HCOOH]$^-$	987.5543, 945.5434, 783.4895, 621.4365, 459.3842, 375.2884	6″–*O*–acetylginsenoside Rg3 or isomer	H	H	L
**M27[a]**	7.40	1193.5950	11.63	[M–H]$^-$	1107.5957, 1089.5858, 915.5332, 783.4906, 621.4375, 459.3845	m–ginsenoside Rb1	H	H	L
**M28**	6.72	1139.5850	8.01	[M–H+HCOOH]$^-$	1093.5798, 961.5369, 781.4744, 619.4211, 475.3834	floralginsenoside P or isomer	L	M	H
**M29**	6.34	977.5325	10.75	[M–H+HCOOH]$^-$	931.5283, 799.4854, 637.4312, 475.3791, 391.2840	notoginsenoside FP1 or isomer	L	L	H
**M30**	6.04	1007.5430	2.69	[M–H+HCOOH]$^-$	961.5380, 781.4744, 635.4165, 491.3735	quinquenoside L2（OT–2Glc–Rha）	H	H	L
**M31**	6.01	841.4939	18.56	[M–H]$^-$	501.3934, 417.2988	notoginsenoside Rt or isomer	L	L	H
**M32**	5.98	1271.6280	7.78	[M–H+HCOOH]$^-$	1225.6199, 1093.5779, 961.5310, 799.4845, 781.4729, 637.4304, 475.3764	isomer of chikusetsusaponin LM6	L	L	H
**M33**	5.89	977.5309	10.08	[M–H]$^-$	879.3737, 765.4410, 609.2709, 547.0343, 375.2896	ginsenoside I or isomer	M	L	H
**M34**	5.86	1163.5850	12.33	[M–H]$^-$	1119.5961, 945.5440, 783.4895, 621.4376, 459.3840	isomer of m–ginsenoside Rc	M	L	H
**M35**	5.83	1153.6000	11.18	[M–H+HCOOH]$^-$	1107.5946, 945.5428, 783.4906, 621.4379, 459.3837, 375.2890	ginsenoside Rb1 or isomer	M	L	H
**M36[a]**	5.82	829.4952	17.89	[M–H]$^-$	701.4215, 621.4369, 459.3813, 375.3023	ginsenoside F2	H	M	L
**M37[a]**	5.62	977.5325	5.49	[M–H+HCOOH]$^-$	801.3646, 669.5836, 553.3723	notoginsenoside Fp1	H	H	L
**M38**	5.59	1139.5840	7.34	[M–H+HCOOH]$^-$	1093.5805, 961.5375, 799.4851, 637.4316, 475.3798, 391.2830	floralginsenoside P or isomer	H	L	L
**M39**	5.18	699.4326	6.96	[M–H+HCOOH]$^-$	513.1210, 459.2246	ginsenoside M7cd or isomer	H	L	L

**M40**	5.14	1139.5850	8.43	[M–H+HCOOH]$^{-}$	1093.5804, 961.5397, 799.4846, 781.4745, 637.4311, 475.3790	floralginsenoside P or isomer	L	M	H
**M41**[a]	5.03	699.4323	9.72	[M–H+HCOOH]$^{-}$	349.2518, 329.1897, 217.1589	pseudoginsenoside Rt5 (24*R*)	L	H	L
**M42**[a]	5.00	945.5426	14.17	[M–H]$^{-}$	765.4783, 605.4077, 537.3428, 457.3662, 373.3141	ginsenoside Rd	H	M	L

[a]: The components identified by comparison with the reference standards.
